# Supplementary material for: Barriers and facilitators to reporting deaths following Ebola surveillance in Sierra Leone: implications for sustainable mortality surveillance based on an exploratory qualitative assessment
Source: BMJ Open. 2021 May 12;11(5):e042976. doi: 10.1136/bmjopen-2020-042976 (PMC8126305; doi:10.1136/bmjopen-2020-042976)
Supplement: Supplementary data [file bmjopen-2020-042976supp001.pdf]

Barriers and facilitators to death reporting following Ebola surveillance in Sierra Leone: Implications for sustainable mortality surveillance

Supplemental Material: Interview Guide

Part A: Community Level Practices and Perceptions

1. **What usually happens after someone dies in this community (who does what/when/how)?**
   - Probe: What are the most important things to do immediately after someone dies?
   - Probe: How is the funeral usually handled / who participates?
   - Probe: How is the burial usually handled / who participates?
   - Probe: Who is notified of the death, and when and how are they notified?
   - Probe: To what extent does the type of death influence how the funeral/burial is handled, and who is informed about it?

2. **Who conducts/helps with the funeral/burial?**
   - Probe: Please tell us why these particular people engaged

3. **Overall, what is the current practice for reporting deaths to authorities (local, district, national 1-1-7) in this community?**
   - Probe: What types of deaths are supposed to be reported?
   - Probe: When should the death be reported, and to whom?
   - Probe: How should the death be reported?
   - Probe: What usually happens after the death is reported?

4. **What usually discourages people from reporting the death of a loved one or neighbor?**
   - Probe: is there any type of death that people are inclined not to report? What are the reasons for this?
   - Further probe for:
     - Time of death
     - Burial delay
     - Age of the deceased
     - Religious reasons
     - Other
   - Probe: What questions do you have for authorities about reporting deaths?

5. **What would encourage or motivate you to report the death of a loved one or neighbor to 117 in the future?**
   - Probe: What types of deaths are you most likely to report? What are the reasons for this?
   - Probe: What should be done differently to improve reporting?
   - Probe: What new things would you like to see happen?
   - Probe: Who can help improve death reporting in this community, if any?

Part B: Personal Experience and Perceptions

Thanks for your responses. I appreciate your openness and willingness to discuss these issues. You already told you us that you did not report the recent death of your loved one. Now, I would like to learn more about that particular situation.

Barriers and facilitators to death reporting following Ebola surveillance in Sierra Leone: Implications for sustainable mortality surveillance

6. **Please describe for me the circumstances surrounding the death of your loved one.**
   - Probe: What was their health situation in the months leading up to their death?
   - Probe: What signs/symptoms were reported or shown leading to the death?
   - Probe: Were there suspicious aspects of the illness? If so, please describe them.
   - Probe: What type of care had they received (traditional vs medical, etc)?
   - Probe: Where and when did the death occur?

7. **Please describe for me the steps that you or the family took after the death:**
   - Probe: What did you or the family first do? Why was this important?
   - Probe: Who did you inform about the death? And how were they informed?
   - Probe: How long did it take from time of death to the time of burial?

8. **Tell us more about burial processes:**
   - Probe: Did you get permission for the burial? If so, how and from whom did you get approval to carry out the burial?
   - Probe: Where was the body buried (public site vs private vs family land)?
   - Probe: What ceremonies were conducted during the burial? (*note to interviewer: don't ask about any specific secrete ceremonies or ritual*)
   - Probe: Who participated in the burial? Why were their participation important?

9. **When you hear "1-1-7" what comes to mind?**
   - Probe: What are your opinions of 117?
   - Probe: Do you think this number should be kept or change?
   - Probe: Please tell us why it should be kept or changed.
   - Probe: How can the 117 system be improved?

10. **Please tell me the reason why you decided not to report the death to the 1-1-7 National Call Center:**
    - Probe: Understanding of government requirements to report all deaths to 117 (or lack thereof)?
    - Probe: Concerns about funeral/burial delays?
    - Probe: Nature or circumstances of the death?
      - Accident and pregnancy related
      - 'Natural causes'
      - Death that resembles Ebola or Lassa
      - Age of the deceased

11. **Would you consider reporting the death of a loved one to 117 in future?**
    - Probe: If so please tell us what may have changed since the present case
    - Probe: What types of deaths are you most likely to report? What are the reasons for this?
    - Probe: What should be done differently?
    - Probe: What new things would you like to see happen?
    - Probe: Who can help improve death reporting in this community?

Barriers and facilitators to death reporting following Ebola surveillance in Sierra Leone: Implications for sustainable mortality surveillance
