# Supplementary material for: Barriers and facilitators to reporting deaths following Ebola surveillance in Sierra Leone: implications for sustainable mortality surveillance based on an exploratory qualitative assessment
Source: BMJ Open. 2021 May 12;11(5):e042976. doi: 10.1136/bmjopen-2020-042976 (PMC8126305; doi:10.1136/bmjopen-2020-042976)
Supplement: Author's manuscript [file bmjopen-2020-042976.draft_revisions.pdf]

BMJ Open is committed to open peer review. As part of this commitment we make the peer review history of every article we publish publicly available.

When an article is published we post the peer reviewers' comments and the authors' responses online. We also post the versions of the paper that were used during peer review. These are the versions that the peer review comments apply to.

The versions of the paper that follow are the versions that were submitted during the peer review process. They are not the versions of record or the final published versions. They should not be cited or distributed as the published version of this manuscript.

BMJ Open is an open access journal and the full, final, typeset and author-corrected version of record of the manuscript is available on our site with no access controls, subscription charges or pay-per-view fees (http://bmjopen.bmj.com).

If you have any questions on BMJ Open's open peer review process please email info.bmjopen@bmj.com

Barriers and facilitators to death reporting following Ebola surveillance in Sierra Leone: Implications for sustainable mortality surveillance

Journal:	*BMJ Open*
Manuscript ID	bmjopen-2020-042976
Article Type:	Original research
Date Submitted by the Author:	20-Jul-2020
Complete List of Authors:	Jalloh, Mohamed; Centers for Disease Control and Prevention, Division of Global Health Protection<br>Kinsman, J; Umea University<br>Conteh, James; Independent Consultant<br>Kaiser, Reinhard; CDC-Sierra Leone<br>Jambai, Amara; Ministry of Health and Sanitation<br>Ekström, Anna; Karolinska Institutet Department of Public Health Sciences<br>Bunnell, Rebecca ; Centers for Disease Control and Prevention<br>Nordenstedt , Helena; Karolinska Institutet Department of Public Health Sciences,
Keywords:	Public health < INFECTIOUS DISEASES, Epidemiology < TROPICAL MEDICINE, QUALITATIVE RESEARCH

SCHOLARONE™
Manuscripts

I, the Submitting Author has the right to grant and does grant on behalf of all authors of the Work (as defined in the below author licence), an exclusive licence and/or a non-exclusive licence for contributions from authors who are: i) UK Crown employees; ii) where BMJ has agreed a CC-BY licence shall apply, and/or iii) in accordance with the terms applicable for US Federal Government officers or employees acting as part of their official duties; on a worldwide, perpetual, irrevocable, royalty-free basis to BMJ Publishing Group Ltd ("BMJ") its licensees and where the relevant Journal is co-owned by BMJ to the co-owners of the Journal, to publish the Work in this journal and any other BMJ products and to exploit all rights, as set out in our licence.

The Submitting Author accepts and understands that any supply made under these terms is made by BMJ to the Submitting Author unless you are acting as an employee on behalf of your employer or a postgraduate student of an affiliated institution which is paying any applicable article publishing charge ("APC") for Open Access articles. Where the Submitting Author wishes to make the Work available on an Open Access basis (and intends to pay the relevant APC), the terms of reuse of such Open Access shall be governed by a Creative Commons licence – details of these licences and which Creative Commons licence will apply to this Work are set out in our licence referred to above.

Other than as permitted in any relevant BMJ Author's Self Archiving Policies, I confirm this Work has not been accepted for publication elsewhere, is not being considered for publication elsewhere and does not duplicate material already published. I confirm all authors consent to publication of this Work and authorise the granting of this licence.

For peer review only

Barriers and facilitators to death reporting following Ebola surveillance in Sierra Leone: Implications for sustainable mortality surveillance

Mohamed F. Jalloh[1,2], John Kinsman[1,3], James Conteh[4], Reinhard Kaiser[2], Amara Jambai[5], Anna Mia Ekström[1], Rebecca E. Bunnell[2], Helena Nordenstedt[1]

1. Department of Global Public Health, Karolinska Institutet, Stockholm, Sweden
2. Division of Global Health Protection, Centers for Disease Control and Prevention, Atlanta, USA
3. Department of Epidemiology and Global Health, Umeå University
4. Independent consultant, Freetown, Sierra Leone
5. Ministry of Health and Sanitation, Freetown, Sierra Leone

Corresponding Author
Mohamed F. Jalloh. Department of Global Public Health, Karolinska Institutet, Stockholm, Sweden. Email: mohamed.jalloh@ki.se. Phone: +1-404-401-2773.

Keywords
Mortality surveillance, death reporting, Ebola, EVD, outbreak, CRVS, Sierra Leone

For peer review only

Abstract

Objectives: To understand the barriers contributing to the sharp decline in the number of deaths (of all causes) reported to a national tollfree telephone line (1-1-7) after the 2014-2016 Ebola outbreak ended in Sierra Leone and explore opportunities for improving routine death reporting as part of a nationwide mortality surveillance system.

Design: An exploratory qualitative assessment comprising 32 in-depth interviews. All interviews were audio-recorded, transcribed, and analysed using content analysis to identify themes.

Setting: Participants were selected from urban and rural communities in two districts that experienced varying levels of Ebola cases during the outbreak. All interviews were conducted in in August 2017 in the post-Ebola-outbreak context in Sierra Leone when the Sierra Leone Ministry of Health and Sanitation was continuing to mandate reporting of all deaths.

Participants: Family members of deceased persons whose deaths were not reported to the 1-1-7 system.

Results: Death reporting barriers were driven by the lack of awareness to report all deaths, lack of services linked to reporting, negative experiences from the Ebola outbreak including prohibition of traditional burial rituals, perception that inevitable deaths do not need to be reported, and situations where prompt burials may be needed. Facilitators of future willingness to report deaths were largely influenced by the perceived communicability and severity of the disease, unexplained circumstances of the death that need investigation, and the potential to leverage existing death notification practices through local leaders.

Conclusions: The findings demonstrate the need to incentivize death reporting behaviours in a post-Ebola outbreak setting. Social mobilization and risk communication efforts may be needed to help the public understand the importance and benefits of sustained and ongoing death reporting. Localized practices for informal death notification through community leaders could be integrated into the formal reporting system to capture community-based deaths that may otherwise be missed.

For peer review only - http://bmjopen.bmj.com/site/about/guidelines.xhtml

Article summary

Strengths and limitations of this study

- This large qualitative assessment helps to explain the complex reasons for the sharp and persistent decline in death reporting levels in Sierra Leone following the 2014-2016 Ebola outbreak.
- The assessment generated novel understanding of barriers and facilitators related to death reporting with themes that may be transferrable to other post-Ebola-outbreak contexts.
- Given that mortality surveillance is a key approach for identifying existing and new public health threats, the findings can help inform strategies for engaging community members to improve death reporting level.
- It is possible that some respondents may have provided socially desirable responses in terms of facilitators to report in order to match previously heard messages during the Ebola outbreak.
- Other key stakeholders with relevant views on mortality surveillance (e.g. health workers and local officials) were not interviewed. Nevertheless, this assessment shed light on the perspectives of family members who failed to report the deaths of their loved ones as mandated by the Government.

only

For peer review only - http://bmjopen.bmj.com/site/about/guidelines.xhtml

Introduction

A popular traditional healer in a remote village in Kailahun district in Sierra Leone became ill and suddenly died around April 30th 2014 after treating patients from neighbouring Guinea.[1] The Sierra Leone Ministry of Health and Sanitation (MoHS) subsequently confirmed an outbreak of Ebola Virus Disease (Ebola) on May 25th 2014, which was linked to the burial of the traditional healer in Kailahun district.[2] Ebola cases quickly spread to neighbouring Kenema district, making the Eastern region the initial epicentre of the outbreak in Sierra Leone.[3] The epicentre of the outbreak shifted from the Eastern region to the Western and Northern regions by September 2014. Over 14,000 people became infected in the country, of whom nearly 4,000 died by the time the outbreak was declared over in November 2015 by the World Health Organization (WHO).[2]

Traditional burials that involved various forms of physical contact with infected corpses were identified as core transmission amplifiers of the Ebolavirus.[4] It has been estimated that nearly three new Ebola cases resulted from every unsafe traditional burial during the outbreak in West Africa.[4] Containing the outbreak required prompt identification of all deaths to ensure safe burial by teams trained on Ebola infection prevention and control.[5] Social mobilization and risk communication efforts were implemented nationally and intensified in high-transmission districts to persuade communities to report all deaths to a national tollfree telephone line using a short dialling code, 1-1-7.[6 7]

Mortality surveillance is a key approach for identifying and responding to public health threats.[8-11] It is used to count the excess number of deaths due to an emerging health threat,[12] describe patterns in mortality occurrence,[8 9] and help to quantify causes of death in a population.[11 13] Mortality surveillance systems have an important role in routine surveillance[8 9] and in health emergency contexts such as during disease outbreaks.[10 12 14] Death reporting through the 1-1-7 system constituted a critical component of identifying and responding to occurrences of deaths in communities to prevent unsafe traditional burials during the Ebola outbreak in Sierra Leone.[14]

Previous analyses of monthly reports of call volumes to the 1-1-7 system showed a substantial decline across all districts after announcing the end of the outbreak.[14 15] In the last year of the outbreak, the average number of deaths reported monthly to the 1-1-7 system fell from approximately 9,000 during January—October 2015, to 4,000 during the enhanced surveillance period (November 2015 to June 2016). The decline continued and reached less than 1,000 reported deaths per month in 2017.[15] A national telephone survey was conducted in April 2017 to investigate the motivations of those who continued to report deaths to the 1-1-7 system after Ebola outbreak ended.[15] Results from the survey showed that people who reported deaths were more motivated to do so when Ebola-like symptoms were present in the deceased. However, since the survey only

For peer review only - http://bmjopen.bmj.com/site/about/guidelines.xhtml

targeted individuals who had reported a death, barriers related to reporting among those who failed to report to the 1-1-7 system were not understood. In this paper we examine death reporting barriers and explore opportunities for improving routine mortality surveillance in the aftermath of the Ebola outbreak in Sierra Leone. The study's findings can help inform mortality surveillance not only in Sierra Leone but also in other in post public health emergency settings.

Methods

In-depth qualitative interviews were conducted with 32 purposefully selected individuals who experienced a death in their household in April-August 2017 but did not report the death to the 1-1-7 system as required by the MoHS. Interviews were conducted by trained native speakers in two districts, one urban: Western Area (n=16) and one rural: Kenema district (n=16). Audio-recordings of the interviews were transcribed and the textual data were analysed using qualitative content analysis.[16] COREQ guidelines are used to describe the methods of our qualitative exploratory assessment.[17]

Setting

The initial purpose of the system when it was established in 2013 was to get feedback from communities on the government's Free Health Care Initiative that provides essential primary health care services at no-cost to children under five years old, pregnant women, and lactating mothers.[18] In August 2014, as Ebola cases began to surge, the Government repurposed the 1-1-7 line for communities to report suspected Ebola cases with a mandate that all deaths, even if not suspected of Ebola, must be reported for safe burial.[14] A national call center was set up for triaging the call alerts to district personnel who dispatched ambulance and burial teams.[19] Alpren et al. have comprehensively documented the implementation of the 1-1-7 system.[14]

The normalization of death reporting in Sierra Leone during the Ebola outbreak presented a unique opportunity for the country to leverage the 1-1-7 system as a foundation for strengthening civil registration of vital statistics (CRVS) in the post-outbreak context. CRVS is part of global efforts to register all births and deaths occurring in all countries.[20] The 2015-2024 global strategic plan for CRVS aims to have functional country systems to record all deaths so that the WHO International Classification of Disease and Injuries can be used to as "the global standard for classifying causes of death in a comparable manner over time and between populations."[20] Prior to the Ebola outbreak in Sierra Leone, deaths were supposed to be reported to the Office of Births and Deaths through its local district offices as per legal mandate established in the 1983 Births and Deaths Registration Act.[21] The pre-Ebola-outbreak death registration level remains unknown.

For peer review only - http://bmjopen.bmj.com/site/about/guidelines.xhtml

The paper-based CRVS death registration systems were never integrated with the digital platforms used in the 1-1-7 system during and after the outbreak.

The MoHS instituted enhanced surveillance mechanisms after the outbreak was declared over by WHO because it anticipated possible flare-ups of new Ebola cases due to sexual transmission of the Ebola virus by male Ebola survivors, which had been reported in Liberia.[22 23] Also, the outbreak in Guinea had not been declared over by WHO, which meant that possible importation of cases needed to be monitored, especially along border regions.[24] Consequently, the MoHS mandated that all deaths must continue to be reported to the 1-1-7 system to help detect possible flare-up of new Ebola cases. Death investigations during the enhanced surveillance period included buccal swabbing of corpses to test for Ebola and determine the need for safe burial.[14] On June 30th 2016, the MoHS announced the end of enhanced Ebola surveillance.[14] The radio announcement stated that starting in July 2016 all deaths were still required to be reported through the 1-1-7 system, however, only deaths that were suspected of Ebola were required to be investigated by district-based surveillance teams. Buccal swabbing of corpses stopped after enhanced surveillance ended. The MoHS aimed to have the 1-1-7 transitioned into the primary mechanism for death registration and mortality surveillance in Sierra Leone including for health workers to report facility-based deaths.

Sampling

Western Area and Kenema districts were purposefully selected for inclusion in the exploratory assessment. Both districts were chosen because of their epidemiological significance during the 2014-2016 Ebola outbreak in Sierra Leone and to allow for rural-urban variations in the sample. Western Area was selected because it had the highest number of reported Ebola cases and it has a large urban population. Kenema was selected because it was an early epicentre of the outbreak and it has a large rural population. We expected that the sociodemographic characteristics and the different experiences of Ebola in these districts would facilitate in-depth understanding of the range of reporting barriers and possible facilitators of willingness to report in the future. Within each district, we conducted eight interviews in high Ebola burden areas and another eight in low burden communities. Ebola epidemiological data from the MoHS showing estimated case counts at sub-district level guided our selection of communities. For the purpose of the assessment, we defined high Ebola burden as $\geq$ 50 cumulative cases and low Ebola burden as $\leq$ 10 cases per community.

Trained data collection teams worked with community mobilizers to help identify households that had experienced one or more deaths between April and August 2017.

For peer review only - http://bmjopen.bmj.com/site/about/guidelines.xhtml

The mobilizers contacted community leaders including religious leaders to inquire about deaths that occurred in the respective communities during the specified period. Based on the information gathered, the mobilizers and data collection teams visited the households referred by community leaders. For a referred household to be eligible to be included in the assessment, the death must not have been reported to the 1-1-7 system. We also used snowball sampling by asking eligible households to refer data collection teams to other households that may have experienced a death during the same period. All eligible households identified during recruitment agreed to participate in the assessment.

Data collection

Data collectors then continued to follow-up with the identified households to confirm eligibility, explain the purpose of the assessment, and ask for informed consent to participate. Once the eligible household was identified by the local team, informed consent was obtained from the head of the household or next of kin of the deceased. Only one interview was conducted per eligible household, and repeat interviews were not conducted. If the household head or next of kin were both unavailable, the data collection team returned to the household at least one more time before it was considered unreachable and another eligible household was approached. After obtaining informed consent from participants, the team used a structured questionnaire to gather basic demographic information about the respondent. This was followed by administering an in-depth interview using a semi-structured guide that covered two broad domains: community level practices and perceptions regarding the death; and personal experiences and perceptions regarding the death (Supplemental Material). The interview guide was pilot tested with a convenience sample of 4 respondents as part of the training of the data collection teams. Feedback from the pilot was used to improve the framing and sequencing of questions and probes.

Data collection was carried out by mixed-gender teams of interviewers and note-takers with extensive prior experience in qualitative data collection in Sierra Leone. All data collection team members were fluent in the predominant local languages of their assigned districts (Krio in Western Area, and Mende or Krio in Kenema), and had post-secondary education in social sciences or public health. All participated in a one-week training that covered informed consent, sampling procedures, best practices for conducting interviews and debriefs, and translation and transcription of audio-recordings. All interviews were audio-recorded with consent from respondents. On average the interviews lasted between 45 and 60 minutes. Interviews were conducted in a secluded area within the vicinity of the home. At the end of each interview, the interviewer and note-taker conducted a short debrief that lasted approximately 30 minutes in order to capture key topics that emerged from the discussion and to document important observations that may help to contextualize the responses. Review of the data and debrief notes indicated

For peer review only - http://bmjopen.bmj.com/site/about/guidelines.xhtml

that analytical data saturation was achieved after 16 interviews were conducted in each district (i.e. when meaningfully new information was no longer emerging from the interviews).

Data management and analysis

The respective teams of interviewers and note-takers translated and transcribed the audio-recordings. Team members conducted peer reviews of each other's transcripts to ensure consistency in translations from local language to English. A supervisor reviewed all transcripts for translation accuracy and fidelity of meaning. However, Interview transcripts were not provided to the participants for their review or correction. The analysis was led by the lead author (MFJ) with support from co-author JC, both of whom are Sierra Leoneans with training and experience in qualitative data analysis. A web-based qualitative software, Dedoose®,[25] was used to support the management and analysis of the data.

An initial set of deductive codes were first generated to reflect meaning units from the questions in semi-structured interview guide. The two analysts (MFJ and JC) generated additional inductive codes from reviewing the transcripts, then proceeded to code the transcripts, independently validated each other's application of codes, and resolved any discrepancies. Textual excerpts were extracted from Dedoose® for each code. The analysts iteratively reviewed, discussed, and interpreted the coded excerpts. The final codes were organized into mutually exclusive sub-categories and categories that reflected latent grouping of concepts. An iterative, interpretative process led to the higher-level grouping of the categories into themes. Preliminary results from the qualitative analysis were presented to stakeholders in Sierra Leone including the MoHS and other surveillance partners. Feedback received from the stakeholder presentation informed our interpretation and framing of the themes.

Patient and public involvement

Patients and/or the public were not involved in the design, conduct, reporting or dissemination plans of this research.

Results

Demographic characteristics of respondents

Out of 32 respondents, 18 (56%) were female, 15 (47%) had no education or only primary school education, 8 (25%) were petty traders, and 22 (69%) self-identified as Muslim. Respondents' age ranged from 27 to 70 years; median age was 38 years. Respondents mostly comprised of relatives of deceased persons (66%), including parents (n=12), spouses (n=5), or children (n=4) (Table 1).

For peer review only - http://bmjopen.bmj.com/site/about/guidelines.xhtml

Summary of themes

Death reporting barriers were driven by the lack of awareness to report all deaths, lack of services linked to reporting, negative experiences from the Ebola outbreak including prohibition of traditional burial rituals, perception that inevitable deaths do not need to be reported, and situations where prompt burials may be needed (Table 2). Facilitators of future willingness to report deaths were largely influenced by the perceived communicability and severity of the disease, unexplained circumstances of the death that need investigation, and the potential to leverage existing death notification practices through local leaders (Table 3).

Barrier 1.1. Lack of awareness to report all deaths

All respondents were unaware that they were required to report the household death after the end of the enhanced Ebola surveillance. Although some respondents knew that the 1-1-7 line was still operational, they thought that only deaths that resembled Ebola needed to be reported.

"… I do not think 1-1-7 is still existing because after Ebola we thought that was the end of 1-1-7. I'm only hearing this from you now. I always listen to the radio, but it has taken a long time I did not hear announcement that when someone dies, we are to call 1-1-7; even in the villages, that is why I did not remember to call 117." – respondent from Western Area district

Barrier 1.2 Lack of reciprocal benefit

Respondents did not see a benefit to report deaths to the 1-1-7 system in a post-Ebola-outbreak setting. The notion of simply notifying 1-1-7 without any associated follow-up action or service was not appreciated by interviewees. In order to report deaths to 1-1-7, respondents said they would expect some follow-up action or service to be provided. For instance, they strongly recommended for prompt ambulance services for sick people and transportation of corpses through 1-1-7. Respondents expressed that such services would help motivate them in the future to use the system.

"…if you take transport, like you take a taxi, to carry a [sick] person to the hospital; when you are going with him and there is traffic, they won't give you way. But let's say you call the 1-1-7 and the 1-1-7 comes with the ambulance, they will be able to give you way because they will take it as an emergency." – respondent from Western Area district

"Then I would like them to give us ambulance in the community so when someone dies, they will be able to take the person and bury him/her quickly." – respondent from Kenema district

For peer review only - http://bmjopen.bmj.com/site/about/guidelines.xhtml

Barrier 1.3 Perceived inevitability of certain deaths

Respondents consistently expressed that they would not consider reporting deaths that they perceive to be inevitable due to age, God's will, minor illness, and long-term illness or disability, drowning, or fire. They also mentioned that patients who die in a health facility do not need to be reported because they presumed that it was the responsibility of the health workers to report.

"The illness that affects someone for so long, for example stroke [complications], which leads to death, we will not report such death. For instance, in our community, we had a man by the name of xxx [redacted] who was affected by stroke and had suffered with it for a very long time; his family had tried all forms of medication, but he did not survive. So, with this type of death the chief themselves will just give permission to the people for burial rather than reporting to 1-1-7." - respondent from Kenema district

Barrier 1.4 Needing to bury promptly

There were two main reasons why prompt burial emerged as a barrier to death reporting. Firstly, circumstances of the death influenced perceptions of when the corpse should be buried including the death of young children, someone who has been ill for a long time (despite their age), and someone who died from an accident (e.g. drowning, fire, road accident). Secondly, Muslim respondents emphasized that they need to bury the corpse within 24 hours to honour Islamic requirements; they feared that reporting may result in burial delay based on their experiences from the Ebola outbreak. One respondent gave an example of how same-day burial was done as per Islamic tradition:

"I went straight to the Imams [at the local mosque] and notified them [of the death]. The Imam came and asked us to take the corpse to the Mosque. The corpse laid there until around twelve o'clock when they washed and wrapped it with Kasankay [white] cloth. We waited for the time … that is two o'clock, then we went to bury him. After the burial the 'Jamat' [mosque congregation] met here [at our house], ate and prayed for him before everyone went back to their homes" – respondent from Western Area district

Barrier 1.5 Negative experiences from the Ebola outbreak

The 1-1-7 system was intricately linked to its widespread use during the Ebola outbreak in Sierra Leone and could not be separated by respondents' negative experiences with how some deaths were handled by burial teams during the outbreak.

"The family would not get access to the corpse or even go close to it. So this knowledge had existed within people that 1-1-7 is not a call to make in order to get help in the burial

For peer review only - http://bmjopen.bmj.com/site/about/guidelines.xhtml

of their loved ones. They will only come to do whatever they feel like, whether it is in a respectful way or not; they don't care. So, with that, people in the community do not favour the 1-1-7. Because how we expect them to bury our loved ones it's the opposite [that they will do]." – respondent from Kenema district

Most participants cited that burial methods used to bury their loved ones during the Ebola outbreak discouraged them from reporting to 1-1-7.

"This 1-1-7 line…I don't want it. I want us to be respecting the [dead] people because the 1-1-7 was not burying our people properly. So, we are burying our people. Let government leave it [burial] up to us. If a doctor check the body [that's fine], but don't let the 1-1-7 - come here until we have buried the corpse" – respondent from Kenema district

Dissatisfaction with burial methods was coupled with discontent about delays by burial teams when they were responding to death alerts during the Ebola outbreak.

"Like during the Ebola time if you call…they [burial teams] will not come [on time]. They will abandon them for some time before coming to take the person. All they care about is for the people to call them... They need to put more efforts into how they respond and treat the people with respect" – respondent from Western Area district

Fear of being quarantined was also mentioned as a barrier for reporting deaths to 1-1-7 as well as the sounds made by the ambulances and spraying of the house with chlorine, which were all associated with Ebola-related stigma. Respondents expressed that just the thought of 1-1-7 alone would usually bring back bitter memories of Ebola.

"The first time I heard about 1-1-7 was during Ebola and each time I hear about 1-1-7 I think about Ebola at once. The moment they talk about 1-1-7, it's a worry for me because during that period, when people see 1-1-7 coming, everybody would run away. When they come to a place, they will spray chlorine all over and everybody avoided body contact like nobody's business, and that worried us too much. That is why we hardly forget about 1-1-7" – respondent from Western Area district

Facilitator 1.1 Presence of Ebola-like symptoms

Knowledge gained during the Ebola outbreak influenced respondents' perceptions of the deaths that should be reported to the 1-1-7 system after the outbreak ended. Participants expressed that they would report a death if it resembled Ebola or Lassa fever, especially in situations where the person bled before dying.

For peer review only - http://bmjopen.bmj.com/site/about/guidelines.xhtml

"For any death pertaining to what government told us [we need to report]. That of a bad disease like Ebola, Lassa fever…The people around will not even dare to touch the person, because it is a transferable disease and it is more common in Eastern Province." – respondent from Kenema district

Facilitator 1.2 Sudden and unexplained deaths

Sudden and unexplained deaths wherein the person was not previously sick were perceived as needing to be reported to the authorities for further investigations.

"Like I said before, when someone dies abruptly, and nothing was wrong with him [before dying]. I will just be looking at him, I will not have the knowledge to know the cause of death, I will not have the machine to show that this is the sickness that caused the death or whether he just fell down and died or whether he just sat down and died. When you go to a medical person [through 1-1-7], he can confirm that this is the cause of the death. If the doctor has confirmed that for real he has died, what can I do?" - respondent from Western Area district

Facilitator 1.3 Existence of other local reporting mechanisms

Informing local leaders—such as religious leader, chiefs, and village headmen—about the death was a common practice that most respondents mentioned. In Kenema, some participants cited that informing local and traditional heads granted them permission for burial without needing to report to the 1-1-7 system. The reporting of these deaths to only local leaders showed that respondents were generally willing to report the deaths but only did so in localized ways outside of the 1-1-7 system.

"Well you will have to go and say to the chief or authority that someone has died amongst us. The chief will ask what happened to that person. The chief will ask you… and you will say [something like] it was a cold, or after two three days I noticed that this person was ill. God has taken his life. This is the way he died. The chief will ask you; and you should answer. You have the chance to report to the police station. You have the chance to report to the chief. And you have the chance to call the family members" – respondent from Kenema district

Discussion

In the aftermath of the Ebola outbreak in Sierra Leone, we identified barriers that prevented people from reporting deaths to the authorities, and we also explored facilitators that would encourage death reporting as part of routine mortality surveillance. Barriers uncovered in our assessment were linked to a lack of awareness to continue reporting all deaths after enhanced Ebola surveillance ended as well as the lack of reporting benefits. Respondents were unaware of the requirement to continue reporting

For peer review only - http://bmjopen.bmj.com/site/about/guidelines.xhtml

all deaths after the enhanced surveillance period, and they were under the impression that only deaths resembling Ebola should be reported to the 1-1-7 system. Consequently, other deaths that were not suspected of Ebola were not reported to the 1-1-7 system. A separate assessment found that after the outbreak ended, people were more motivated to report deaths to the 1-1-7 system if Ebola-like symptoms were present in the decedent.[15] Our findings further demonstrated that although respondents did not report deaths through the 1-1-7 system as mandated by their government, they informed local councils and local leaders about the deaths. Therefore, integrating localized practices for death reporting into routine surveillance mortality systems may help optimize the number of deaths captured. Respondents complained about the lack of any reporting benefits associated with death reporting in a post-Ebola-outbreak context. They wanted ambulance services to be linked to 1-1-7 reporting as done during the outbreak. Contextually, past experiences from the Ebola outbreak served as both facilitators and barriers. Past outbreak experiences reinforced the importance of reporting when Ebola is suspected to avoid transmission risks. On the other hand, past experiences that involved dissatisfaction with how burials were handled during the outbreak may have discouraged reporting to the 1-1-7 system.

Despite efforts to promote reporting of all deaths during the outbreak, communities continued to express dissatisfaction with how their loved ones were buried and there were instances of secret burials that occurred outside of the safe burial process, which may have been due to discontent with safe burials or wanting to comply with secret society practices.[26 27] Although dissatisfaction persisted regarding Ebola safe burial processes, communities were willing to comply with reporting because they wanted to help end Ebola transmission in the country.[28] In the waning period of the Ebola outbreak in 2015, an assessment of the community event-based surveillance showed that over 12,000 reports were submitted and investigated, out of which 287 met case-definition for suspected Ebola and 16 were confirmed positive for Ebola.[29] In that assessment, it was revealed that community event-based surveillance detected four new Ebola cases that were not epidemiologically linked and could have gone undetected.

The Ebola outbreak was tragic in many ways as demonstrated by the thousands of lives lost and the unquantifiable suffering inflicted upon the people of Sierra Leone. The pain and misery they endured was evident in our assessment when they talked about their experiences during the outbreak. Even though people were sometimes dissatisfied with how the burials were handled or delayed, they recognized that it was to their benefit to have a safe burial to avoid household transmission risk. Nevertheless, normalizing death reporting during the outbreak required gaining the trust of communities by engaging them to appreciate the benefits of reporting. The halting of ambulance services after the Ebola

For peer review only - http://bmjopen.bmj.com/site/about/guidelines.xhtml

outbreak ended prompted people to question why those services were only provided during the outbreak response.

The principle of *positive reciprocity* has been well established in social psychology,[30-32] which implies that people become motivated to comply with a request when they receive something in-return for their action. This notion is also supported in the health behaviour literature. For instance, applications of the Health Belief Model have shown that the perceived benefit associated with a behaviour is a strong predictor of engaging in the behavior.[33 34] Our findings are consistent with notions of reciprocity and perceived benefit such that respondents expressed that they will be willing to report deaths if tangible benefits are provided for complying (e.g. ambulance services).

The normalization of death reporting during the outbreak was a new opportunity for Sierra Leone to establish CRVS after the outbreak ended. Realizing this potential for a functional CRVS system[35] would require a strong social mobilization component to ensure optimal community-based reporting. It has been documented that surveillance and laboratory systems from the Ebola outbreak in West Africa served as a springboard for strengthening health systems as part of global health security efforts to prevent, detect, and respond to disease threats.[36] Sierra Leone was able to leverage Ebola surveillance infrastructure to strengthen Integrated Disease Surveillance and Response for epidemic-prone diseases.[37] The same could be done for transitioning the 1-1-7 system into a routine mortality surveillance tool.

Limitations

The exploratory qualitative assessment is subject to several limitations. The results are not generalizable beyond the 32 individuals interviewed. While this is an inherent limitation to most qualitative research, it is important to note that we never intended to produce generalizable results. Instead, our aim was to generate in-depth understanding of barriers and facilitators of death reporting with themes that may be transferrable to other local contexts. It is possible that some respondents may have provided socially desirable responses in terms of facilitators to report in order to match previously heard messages during the Ebola outbreak. Because theoretical sampling was not used, as done in grounded theory approaches for example,[38] other individuals outside of family members (e.g. health workers, local city council officials) were not interviewed. Additional research with more diverse stakeholder may help shed light on additional barriers and facilitators.

Conclusion

Respondents misunderstood the *policy of reporting all deaths* after the end of enhanced Ebola surveillance in Sierra Leone, which may have been due to communication gaps in

For peer review only - http://bmjopen.bmj.com/site/about/guidelines.xhtml

the government's official announcement of the mandate. We found that respondents perceived that only suspected Ebola deaths should be reported to the 1-1-7 system. The lack of awareness to *report all deaths* and the lack of perceived reporting benefits were the main reasons for failure to report the deaths. Given that Sierra Leone is now several years removed from the Ebola outbreak, our results reinforce the need to review its death reporting policy to make sure it is aligned with the post-outbreak context where prevention of Ebola transmission may no longer sufficiently motivate people to report deaths in their household as was done during the outbreak. The post-outbreak death reporting policy should consider integrating tangible benefits for those who report. We found that ambulance services may help incentivize people to report while also minimizing any disruptions to the traditional burial processes. Existing practices for informal death notification through local leaders should also be leveraged to capture community-based deaths that may be missed by the formal reporting system. Improving routine death reporting may be supported by well-planned social mobilization efforts to educate communities about the death reporting policy, promote the reporting benefits, and facilitate optimal compliance.

Acknowledgements
We thank the 32 participants for the invaluable information they provided us under difficult circumstances. This paper is dedicated to the memories of the loved ones.

Contributors
MFJ led the conceptualization and design of the assessment with contributions from JK, RK, and HN. MFJ trained and supervised data collectors. MFJ analysed the data with guidance from JK and HN. All co-authors contributed to the interpretation of the results. MFJ led the writing of the manuscript with contributions from all co-authors. The final version of the manuscript was approved by all co-authors.

Funding
Data collection for this work was supported by U.S. Centers for Disease Control and Prevention through a cooperative agreement with eHealth Africa.

Competing interest
The authors have none to declare.

Data availability
All relevant data are presented. No additional data available.

Ethics approval
The assessment was approved by the Sierra Leone MoHS as a routine public health activity. In addition, the Center for Global Health at the U.S. Centers for Disease Control

For peer review only - http://bmjopen.bmj.com/site/about/guidelines.xhtml

and Prevention approved the assessment as a program evaluation activity (CGH HSR Tracking # 2017-327).

Disclaimer

The findings and conclusions in this report are those of the authors and do not necessarily represent the official position of the U.S. Centers for Disease Control and Prevention, Karolinska Institutet, eHealth Africa, or Sierra Leone Ministry of Health and Sanitation.

References

1. Wauquier N, Bangura J, Moses L, et al. Understanding the emergence of Ebola virus disease in Sierra Leone: Stalking the virus in the threatening wake of emergence. *PLoS Curr* 2015;7.

2. World Health Organization. Ebola situation reports, 2016. Available: http://apps.who.int/ebola/ebola-situation-reports [Accessed November 27 2017].

3. Senga M, Koi A, Moses L, et al. Contact tracing performance during the Ebola virus disease outbreak in Kenema district, Sierra Leone. *Philos Trans R Soc Lond B Biol Sci* 2017;372(1721).

4. Tiffany A, Dalziel BD, Kagume Njenge H, et al. Estimating the number of secondary Ebola cases resulting from an unsafe burial and risk factors for transmission during the West Africa Ebola epidemic. *PLoS neglected tropical diseases* 2017;11(6):e0005491.

5. Frieden TR, Damon IK. Ebola in West Africa--CDC's Role in Epidemic Detection, Control, and Prevention. *Emerg Infect Dis* 2015;21(11):1897-905.

6. Bedson J, Jalloh MF, Pedi D, et al. Community engagement in outbreak response: lessons from the 2014-2016 Ebola outbreak in Sierra Leone. *BMJ Glob Health* 2020.

7. Pedi D, Gillespie A, Bedson J, et al. The Development of Standard Operating Procedures for Social Mobilization and Community Engagement in Sierra Leone During the West Africa Ebola Outbreak of 2014-2015. *J Health Commun* 2017;22(sup1):39-50.

8. Liu S, Wu X, Lopez AD, et al. An integrated national mortality surveillance system for death registration and mortality surveillance, China. *Bulletin of the World Health Organization* 2016;94(1):46-57.

For peer review only - http://bmjopen.bmj.com/site/about/guidelines.xhtml

9. Krishnan A, Das DK. Mortality surveillance in India: Past, present, and future. *Indian journal of public health* 2019;63(3):163-64.

10. Setel P, AbouZahr C, Atuheire EB, et al. Mortality surveillance during the COVID-19 pandemic. *Bulletin of the World Health Organization* 2020;98(6):374.

11. Taylor AW, Blau DM, Bassat Q, et al. Initial findings from a novel population-based child mortality surveillance approach: a descriptive study. *Lancet Glob Health* 2020;8(7):e909-e19.

12. Michelozzi P, de'Donato F, Scortichini M, et al. Mortality impacts of the coronavirus disease (COVID-19) outbreak by sex and age: rapid mortality surveillance system, Italy, 1 February to 18 April 2020. *Euro Surveill* 2020;25(19).

13. Anteneh A, Endris BS. Injury related adult deaths in Addis Ababa, Ethiopia: analysis of data from verbal autopsy. *BMC Public Health* 2020;20(1):926.

14. Alpren C, Jalloh MF, Kaiser R, et al. The 117 call alert system in Sierra Leone: from rapid Ebola notification to routine death reporting. *BMJ Glob Health* 2017;2(3):e000392.

15. Jalloh MF, Kaiser R, Diop M, et al. National reporting of deaths after enhanced Ebola surveillance in Sierra Leone Freetown: Sierra Leone Ministry of Health and Sanitation, 2020.

16. Graneheim UH, Lundman B. Qualitative content analysis in nursing research: concepts, procedures and measures to achieve trustworthiness. *Nurse education today* 2004;24(2):105-12. doi: 10.1016/j.nedt.2003.10.001

17. Tong A, Sainsbury P, Craig J. Consolidated criteria for reporting qualitative research (COREQ): a 32-item checklist for interviews and focus groups. *International journal for quality in health care : journal of the International Society for Quality in Health Care* 2007;19(6):349-57. doi: 10.1093/intqhc/mzm042

18. Witter S, Wurie H, Bertone MP. The free health care initiative: how has it affected health workers in Sierra Leone? *Health policy and planning* 2016;31(1):1-9. doi: 10.1093/heapol/czv006

19. eHealth Africa. eHealth Africa and partners boost Sierra Leone's surveillance capacity through 117 Call Center, 2019. Available: https://www.ehealthafrica.org/117-call-center [Accessed February 5 2019].

For peer review only - http://bmjopen.bmj.com/site/about/guidelines.xhtml

20. World Bank. Global civil registration and vital statistics: Scalign up investments plan 2015-2014. Washington DC: World Bank, 2014.

21. United Nations Economic Commission for Africa. Births and deaths in Sierra Leone, 2012. Available: https://www.uneca.org/sites/default/files/images/birth_and_death-sierra_leone.pdf [Accessed February 5 2019].

22. Deen GF, Broutet N, Xu W, et al. Ebola RNA Persistence in Semen of Ebola Virus Disease Survivors - Final Report. *N Engl J Med* 2017;377(15):1428-37.

23. Christie A, Davies-Wayne GJ, Cordier-Lassalle T, et al. Possible sexual transmission of Ebola virus - Liberia, 2015. *MMWR Morb Mortal Wkly Rep* 2015;64(17):479-81.

24. Diallo B, Sissoko D, Loman NJ, et al. Resurgence of Ebola Virus Disease in Guinea Linked to a Survivor With Virus Persistence in Seminal Fluid for More Than 500 Days. *Clin Infect Dis* 2016;63(10):1353-56. doi: 10.1093/cid/ciw601

25. SocioCultural Research Consultants. Dedoose, 2017. Available: https://www.dedoose.com/ [Accessed September 1 2017].

26. Nielsen CF, Kidd S, Sillah AR, et al. Improving burial practices and cemetery management during an Ebola virus disease epidemic - Sierra Leone, 2014. *MMWR Morb Mortal Wkly Rep* 2015;64(1):20-7.

27. Lee-Kwan SH, DeLuca N, Bunnell R, et al. Facilitators and Barriers to Community Acceptance of Safe, Dignified Medical Burials in the Context of an Ebola Epidemic, Sierra Leone, 2014. *J Health Commun* 2017;22(sup1):24-30.

28. Nuriddin A, Jalloh MF, Meyer E, et al. Trust, fear, stigma and disruptions: community perceptions and experiences during periods of low but ongoing transmission of Ebola virus disease in Sierra Leone, 2015. *BMJ Glob Health* 2018;3(2):e000410.

29. Ratnayake R, Crowe SJ, Jasperse J, et al. Assessment of Community Event-Based Surveillance for Ebola Virus Disease, Sierra Leone, 2015. *Emerg Infect Dis* 2016;22(8):1431-7.

30. Goranson REB, L. Reciprocity and responsibility reactions to prior help. *Journal of Personality and Social Psychology* 1996;3(2):227–32.

For peer review only

For peer review only - http://bmjopen.bmj.com/site/about/guidelines.xhtml

31. Gouldner AW. The norm of reciprocity: a preliminary statement. *American Sociological Review* 1960;25(2).

32. Regan DT. Effects of a favor and liking on compliance. *Journal of Experimental Social Psychology* 1971;7(6):627-39.

33. Vermandere H, van Stam MA, Naanyu V, et al. Uptake of the human papillomavirus vaccine in Kenya: testing the health belief model through pathway modeling on cohort data. *Globalization and health* 2016;12(1):72. doi: 10.1186/s12992-016-0211-7

34. Fall E, Izaute M, Chakroun-Baggioni N. How can the health belief model and self-determination theory predict both influenza vaccination and vaccination intention? A longitudinal study among university students. *Psychology & health* 2018;33(6):746-64.

35. AbouZahr C, de Savigny D, Mikkelsen L, et al. Civil registration and vital statistics: progress in the data revolution for counting and accountability. *Lancet* 2015;386(10001):1373-85.

36. Marston BJ, Dokubo EK, van Steelandt A, et al. Ebola response impact on public healthpPrograms, West Africa, 2014-2017. *Emerg Infect Dis* 2017;23(13).

37. Njuguna C, Jambai A, Chimbaru A, et al. Revitalization of integrated disease surveillance and response in Sierra Leone post Ebola virus disease outbreak. *BMC Public Health* 2019;19(1):364.

38. Moser A, Korstjens I. Series: Practical guidance to qualitative research. Part 3: Sampling, data collection and analysis. *The European journal of general practice* 2018;24(1):9-18.

For peer review only

For peer review only - http://bmjopen.bmj.com/site/about/guidelines.xhtml

TABLES

Table 1. Sociodemographic characteristics of respondents, Sierra Leone, 2017

Characteristic	Number of respondents (N = 32)
**Sex**	
Female	18
Male	14
**Education**	
None or primary only	15
Secondary or higher	17
**Religion**	
Muslim	22
Christian	10
**Occupation**	
Petty trader	8
Skilled labor	7
Private business	4
Teacher	3
Student	3
Unemployed	3
Driver / bike rider	2
Farmer	1
Civil service	1
**Age (years)**	Median = 38
21-30	6
31-40	15
41-50	4
51-60	6
61-70	1

For peer review only

Table 2. Thematic area on barriers to reporting deaths in the aftermath of the Ebola outbreak, Sierra Leone, 2017

Code	Category	Theme
1.1.1 Reporting only required during Ebola	1.1 Lack of awareness to report all deaths	Barriers to reporting deaths in the aftermath of the Ebola outbreak
1.1.2 No more need to report to 1-1-7		
1.1.3 Only Ebola-like deaths should be reported		
1.2.1 No services for people while alive	Lack of reciprocal benefit	
1.2.2 Too much focus on dead people		
1.2.3 Nothing done for sick people		
1.2.4 Nothing happens if you report		
1.2.5 No help with burial		
1.2.6 Just a government line		
1.3.1 Old-age / God's time	1.3 Perceived inevitability of certain deaths	
1.3.2 Minor illness		
1.3.3 Long-term illness or disability		
1.3.4 Death occurred in health facility		
1.4.1 Islamic requirement to bury within 24 hours	1.4 Needing to bury promptly	
1.4.2 Body recovered from drowning		
1.4.3 Body recovered from fire		
1.4.4 Young child		
1.4.5 Sick for long time		
1.5.1 Wanting to forget about 117	1.5 Negative experiences from the Ebola outbreak	
1.5.2 Reporting brings sadness to family		
1.5.3 Painful memory of Ebola		
1.5.4 Burial delays during Ebola		
1.5.5 Restrictions on traditional burials		
1.5.6 Fear of quarantine		
1.5.7 Fear of ambulance		
1.5.8 Line was used during Ebola		
1.5.9 Don't like the number		

Table 3. Thematic area on facilitators of future intention to report deaths, Sierra Leone, 2017

Code	Category	Theme
1.1.1 Resembling Ebola	1.1 Presence of Ebola-like symptoms	Facilitators of willingness to report deaths in the future
1.1.2 Resembling Lassa		
1.1.3 Bleeding before dying		
1.2.1 Sudden death without illness	1.2 Sudden and unexplained death	
1.2.2 Wanting to know cause of death		
1.3.1 Informing chief	1.3 Existence of other local reporting mechanisms	
1.3.2 Informing religious leaders		
1.3.3 Informing elders		
1.3.4 Inform City Council for burial permit		
1.3.5 Informing Office of Births and Deaths for death certificate		

For peer review only - http://bmjopen.bmj.com/site/about/guidelines.xhtml

Barriers and facilitators to death reporting following Ebola surveillance in Sierra Leone: Implications for sustainable mortality surveillance

Supplemental Material: Interview Guide

Part A: Community Level Practices and Perceptions

1. **What usually happens after someone dies in this community (who does what/when/how)?**
   - Probe: What are the most important things to do immediately after someone dies?
   - Probe: How is the funeral usually handled / who participates?
   - Probe: How is the burial usually handled / who participates?
   - Probe: Who is notified of the death, and when and how are they notified?
   - Probe: To what extent does the type of death influence how the funeral/burial is handled, and who is informed about it?

2. **Who conducts/helps with the funeral/burial?**
   - Probe: Please tell us why these particular people engaged

3. **Overall, what is the current practice for reporting deaths to authorities (local, district, national 1-1-7) in this community?**
   - Probe: What types of deaths are supposed to be reported?
   - Probe: When should the death be reported, and to whom?
   - Probe: How should the death be reported?
   - Probe: What usually happens after the death is reported?

4. **What usually discourages people from reporting the death of a loved one or neighbor?**
   - Probe: is there any type of death that people are inclined not to report? What are the reasons for this?
   - Further probe for:
     - Time of death
     - Burial delay
     - Age of the deceased
     - Religious reasons
     - Other
   - Probe: What questions do you have for authorities about reporting deaths?

5. **What would encourage or motivate you to report the death of a loved one or neighbor to 117 in the future?**
   - Probe: What types of deaths are you most likely to report? What are the reasons for this?
   - Probe: What should be done differently to improve reporting?
   - Probe: What new things would you like to see happen?
   - Probe: Who can help improve death reporting in this community, if any?

Part B: Personal Experience and Perceptions

Thanks for your responses. I appreciate your openness and willingness to discuss these issues. You already told you us that you did not report the recent death of your loved one. Now, I would like to learn more about that particular situation.

For peer review only

Barriers and facilitators to death reporting following Ebola surveillance in Sierra Leone: Implications for sustainable mortality surveillance

6. **Please describe for me the circumstances surrounding the death of your loved one.**
    - Probe: What was their health situation in the months leading up to their death?
    - Probe: What signs/symptoms were reported or shown leading to the death?
    - Probe: Were there suspicious aspects of the illness? If so, please describe them.
    - Probe: What type of care had they received (traditional vs medical, etc)?
    - Probe: Where and when did the death occur?

7. **Please describe for me the steps that you or the family took after the death:**
    - Probe: What did you or the family first do? Why was this important?
    - Probe: Who did you inform about the death? And how were they informed?
    - Probe: How long did it take from time of death to the time of burial?

8. **Tell us more about burial processes:**
    - Probe: Did you get permission for the burial? If so, how and from whom did you get approval to carry out the burial?
    - Probe: Where was the body buried (public site vs private vs family land)?
    - Probe: What ceremonies were conducted during the burial? (*note to interviewer: don't ask about any specific secrete ceremonies or ritual*)
    - Probe: Who participated in the burial? Why were their participation important?

9. **When you hear "1-1-7" what comes to mind?**
    - Probe: What are your opinions of 117?
    - Probe: Do you think this number should be kept or change?
    - Probe: Please tell us why it should be kept or changed.
    - Probe: How can the 117 system be improved?

10. **Please tell me the reason why you decided not to report the death to the 1-1-7 National Call Center:**
    - Probe: Understanding of government requirements to report all deaths to 117 (or lack thereof)?
    - Probe: Concerns about funeral/burial delays?
    - Probe: Nature or circumstances of the death?
        - Accident and pregnancy related
        - 'Natural causes'
        - Death that resembles Ebola or Lassa
        - Age of the deceased

11. **Would you consider reporting the death of a loved one to 117 in future?**
    - Probe: If so please tell us what may have changed since the present case
    - Probe: What types of deaths are you most likely to report? What are the reasons for this?
    - Probe: What should be done differently?
    - Probe: What new things would you like to see happen?
    - Probe: Who can help improve death reporting in this community?

For peer review only

Barriers and facilitators to death reporting following Ebola surveillance in Sierra Leone: Implications for sustainable mortality surveillance

For peer review only

COREQ (COnsolidated criteria for REporting Qualitative research) Checklist

A checklist of items that should be included in reports of qualitative research. You must report the page number in your manuscript where you consider each of the items listed in this checklist. If you have not included this information, either revise your manuscript accordingly before submitting or note N/A.

Topic	Item No.	Guide Questions/Description	Reported on Page No.
**Domain 1: Research team and reflexivity**			
Personal characteristics			
Interviewer/facilitator	1	Which author/s conducted the interview or focus group?	
Credentials	2	What were the researcher's credentials? E.g. PhD, MD	
Occupation	3	What was their occupation at the time of the study?	
Gender	4	Was the researcher male or female?	
Experience and training	5	What experience or training did the researcher have?	
Relationship with participants			
Relationship established	6	Was a relationship established prior to study commencement?	
Participant knowledge of the interviewer	7	What did the participants know about the researcher? e.g. personal goals, reasons for doing the research	
Interviewer characteristics	8	What characteristics were reported about the inter viewer/facilitator? e.g. Bias, assumptions, reasons and interests in the research topic	
**Domain 2: Study design**			
Theoretical framework			
Methodological orientation and Theory	9	What methodological orientation was stated to underpin the study? e.g. grounded theory, discourse analysis, ethnography, phenomenology, content analysis	
Participant selection			
Sampling	10	How were participants selected? e.g. purposive, convenience, consecutive, snowball	
Method of approach	11	How were participants approached? e.g. face-to-face, telephone, mail, email	
Sample size	12	How many participants were in the study?	
Non-participation	13	How many people refused to participate or dropped out? Reasons?	
Setting			
Setting of data collection	14	Where was the data collected? e.g. home, clinic, workplace	
Presence of non-participants	15	Was anyone else present besides the participants and researchers?	
Description of sample	16	What are the important characteristics of the sample? e.g. demographic data, date	
Data collection			
Interview guide	17	Were questions, prompts, guides provided by the authors? Was it pilot tested?	
Repeat interviews	18	Were repeat inter views carried out? If yes, how many?	
Audio/visual recording	19	Did the research use audio or visual recording to collect the data?	
Field notes	20	Were field notes made during and/or after the inter view or focus group?	
Duration	21	What was the duration of the inter views or focus group?	
Data saturation	22	Was data saturation discussed?	
Transcripts returned	23	Were transcripts returned to participants for comment and/or	

For peer review only

Topic	Item No.	Guide Questions/Description	Reported on Page No.
		correction?	
**Domain 3: analysis and findings**			
Data analysis			
Number of data coders	24	How many data coders coded the data?	
Description of the coding tree	25	Did authors provide a description of the coding tree?	
Derivation of themes	26	Were themes identified in advance or derived from the data?	
Software	27	What software, if applicable, was used to manage the data?	
Participant checking	28	Did participants provide feedback on the findings?	
Reporting			
Quotations presented	29	Were participant quotations presented to illustrate the themes/findings? Was each quotation identified? e.g. participant number	
Data and findings consistent	30	Was there consistency between the data presented and the findings?	
Clarity of major themes	31	Were major themes clearly presented in the findings?	
Clarity of minor themes	32	Is there a description of diverse cases or discussion of minor themes?	

Developed from: Tong A, Sainsbury P, Craig J. Consolidated criteria for reporting qualitative research (COREQ): a 32-item checklist for interviews and focus groups. *International Journal for Quality in Health Care*. 2007. Volume 19, Number 6: pp. 349 – 357

Once you have completed this checklist, please save a copy and upload it as part of your submission. DO NOT include this checklist as part of the main manuscript document. It must be uploaded as a separate file.

For peer review only

BMJ Open

Barriers and facilitators to death reporting following Ebola surveillance in Sierra Leone: Implications for sustainable mortality surveillance

Journal:	*BMJ Open*
Manuscript ID	bmjopen-2020-042976.R1
Article Type:	Original research
Date Submitted by the Author:	09-Feb-2021
Complete List of Authors:	Jalloh, Mohamed; Karolinska Institutet, Department of Global Public Health; Centers for Disease Control and Prevention, Division of Global Health Protection<br>Kinsman, J; Umea University<br>Conteh, James; Independent Consultant<br>Kaiser, Reinhard; CDC-Sierra Leone<br>Jambai, Amara; Ministry of Health and Sanitation<br>Ekström, Anna; Karolinska Institutet Department of Public Health Sciences<br>Bunnell, Rebecca ; Centers for Disease Control and Prevention<br>Nordenstedt , Helena; Karolinska Institutet Department of Public Health Sciences,
**Primary Subject Heading**:	Public health
Secondary Subject Heading:	Epidemiology, Health policy, Qualitative research
Keywords:	Public health < INFECTIOUS DISEASES, Epidemiology < TROPICAL MEDICINE, QUALITATIVE RESEARCH

SCHOLARONE™
Manuscripts

I, the Submitting Author has the right to grant and does grant on behalf of all authors of the Work (as defined in the below author licence), an exclusive licence and/or a non-exclusive licence for contributions from authors who are: i) UK Crown employees; ii) where BMJ has agreed a CC-BY licence shall apply, and/or iii) in accordance with the terms applicable for US Federal Government officers or employees acting as part of their official duties; on a worldwide, perpetual, irrevocable, royalty-free basis to BMJ Publishing Group Ltd ("BMJ") its licensees and where the relevant Journal is co-owned by BMJ to the co-owners of the Journal, to publish the Work in this journal and any other BMJ products and to exploit all rights, as set out in our licence.

The Submitting Author accepts and understands that any supply made under these terms is made by BMJ to the Submitting Author unless you are acting as an employee on behalf of your employer or a postgraduate student of an affiliated institution which is paying any applicable article publishing charge ("APC") for Open Access articles. Where the Submitting Author wishes to make the Work available on an Open Access basis (and intends to pay the relevant APC), the terms of reuse of such Open Access shall be governed by a Creative Commons licence – details of these licences and which Creative Commons licence will apply to this Work are set out in our licence referred to above.

Other than as permitted in any relevant BMJ Author's Self Archiving Policies, I confirm this Work has not been accepted for publication elsewhere, is not being considered for publication elsewhere and does not duplicate material already published. I confirm all authors consent to publication of this Work and authorise the granting of this licence.

For peer review only

Barriers and facilitators to death reporting following Ebola surveillance in Sierra Leone: Implications for sustainable mortality surveillance based on an exploratory qualitative assessment

Mohamed F. Jalloh[1,2], John Kinsman[1,3], James Conteh[4], Reinhard Kaiser[2], Amara Jambai[5], Anna Mia Ekström[1], Rebecca E. Bunnell[2], Helena Nordenstedt[1]

1. Department of Global Public Health, Karolinska Institutet, Stockholm, Sweden
2. Division of Global Health Protection, Centers for Disease Control and Prevention, Atlanta, USA
3. Department of Epidemiology and Global Health, Umeå University
4. Independent consultant, Freetown, Sierra Leone
5. Ministry of Health and Sanitation, Freetown, Sierra Leone

Corresponding Author
Mohamed F. Jalloh. Department of Global Public Health, Karolinska Institutet, Stockholm, Sweden. Email: mohamed.jalloh@ki.se. Phone: +1-404-401-2773.

Keywords
Mortality surveillance, death reporting, Ebola, outbreak, civil registration of vital statistics, Sierra Leone, West Africa

For peer review only

Abstract

Objectives: To understand the barriers contributing to the more than three-fold decline in the number of deaths (of all causes) reported to a national tollfree telephone line (1-1-7) after the 2014-2016 Ebola outbreak ended in Sierra Leone and explore opportunities for improving routine death reporting as part of a nationwide mortality surveillance system.

Design: An exploratory qualitative assessment comprising 32 in-depth interviews (16 in Kenema district and 16 in Western Area). All interviews were audio-recorded, transcribed, and analysed using content analysis to identify themes.

Setting: Participants were selected from urban and rural communities in two districts that experienced varying levels of Ebola cases during the outbreak. All interviews were conducted in in August 2017 in the post-Ebola-outbreak context in Sierra Leone when the Sierra Leone Ministry of Health and Sanitation was continuing to mandate reporting of all deaths.

Participants: Family members of deceased persons whose deaths were not reported to the 1-1-7 system.

Results: Death reporting barriers were driven by the lack of awareness to report all deaths, lack of services linked to reporting, negative experiences from the Ebola outbreak including prohibition of traditional burial rituals, perception that inevitable deaths do not need to be reported, and situations where prompt burials may be needed. Facilitators of future willingness to report deaths were largely influenced by the perceived communicability and severity of the disease, unexplained circumstances of the death that need investigation, and the potential to leverage existing death notification practices through local leaders.

Conclusions: Social mobilization and risk communication efforts are needed to help the public understand the importance and benefits of sustained and ongoing death reporting after an Ebola outbreak. Localized practices for informal death notification through community leaders could be integrated into the formal reporting system to capture community-based deaths that may otherwise be missed.

For peer review only - http://bmjopen.bmj.com/site/about/guidelines.xhtml

Article summary

Strengths and limitations of this study

- This large qualitative assessment helps to explain the complex reasons for the sharp and persistent decline in death reporting levels in Sierra Leone following the 2014-2016 Ebola outbreak.
- The assessment generated novel understanding of barriers and facilitators related to death reporting with themes that may be transferrable to other post-Ebola-outbreak contexts.
- Given that mortality surveillance is a key approach for identifying existing and new public health threats, the findings can help inform strategies for engaging community members to improve death reporting level.
- It is possible that some respondents may have provided socially desirable responses in terms of facilitators to report in order to match previously heard messages during the Ebola outbreak.
- Other key stakeholders with relevant views on mortality surveillance (e.g. health workers and local officials) were not interviewed. Nevertheless, this assessment shed light on the perspectives of family members who failed to report the deaths of their loved ones as mandated by the Government.

only

For peer review only - http://bmjopen.bmj.com/site/about/guidelines.xhtml

Introduction

A popular traditional healer in a remote village in Kailahun district in Sierra Leone became ill and suddenly died around April 30th 2014 after treating patients from neighbouring Guinea.[1] The Sierra Leone Ministry of Health and Sanitation (MoHS) subsequently confirmed an outbreak of Ebola Virus Disease (Ebola) on May 25th 2014, which was linked to the burial of the traditional healer in Kailahun district.[2] Ebola cases quickly spread to neighbouring Kenema district, making the Eastern region the initial epicentre of the outbreak in Sierra Leone.[3] The epicentre of the outbreak shifted from the Eastern region to the Western and Northern regions by September 2014. Over 14,000 people became infected in the country, of whom nearly 4,000 died by the time the outbreak was declared over in November 2015 by the World Health Organization (WHO).[2]

Traditional burials that involved various forms of physical contact with infected corpses were identified as core transmission amplifiers of the Ebolavirus.[4] It has been estimated that nearly three new Ebola cases resulted from every unsafe traditional burial during the outbreak in West Africa.[4] Containing the outbreak required prompt identification of all deaths to ensure safe burial by teams trained on Ebola infection prevention and control.[5] Social mobilization and risk communication efforts were implemented nationally and intensified in high-transmission districts to persuade communities to report all deaths to a national tollfree telephone line using a short dialling code, 1-1-7.[6 7]

Mortality surveillance is a key approach for identifying and responding to public health threats[8] in both high income countries[9] and low- and middle-income countries[8 10-12]as part of routine surveillance[10 11] as well as in health emergency contexts including during disease outbreaks.[8 9 13] Mortality surveillance systems have been relied upon to count the excess number of deaths due to an emerging health threat,[9] describe patterns in mortality occurrence,[10 11] and help to quantify causes of death in a population.[12 14]Governments' vital registration systems have been used for national mortality surveillance purposes to monitor and describe deaths occurring in a country. In addition, or alternatively, sample-based mortality surveillance systems have been used to generate nationally representative data on deaths. Vital registration systems and sample-based systems have been combined into an integrated mortality surveillance system.[10] In other instances, mortality surveillance systems have focused on sub-population groups (e.g. children) within geographically defined sub-national units.[12]

In Sierra Leone, death reporting through the 1-1-7 system constituted a critical component of identifying and responding to occurrences of deaths in communities to prevent unsafe traditional burials during the Ebola outbreak.[13] Previous analyses of monthly reports of call volumes to the 1-1-7 system showed a substantial decline across all districts after announcing the end of the outbreak.[13 15] In the last year of the outbreak, the average

For peer review only - http://bmjopen.bmj.com/site/about/guidelines.xhtml

number of deaths reported monthly to the 1-1-7 system fell from approximately 9,000 during January—October 2015, to 4,000 during the enhanced surveillance period (November 2015 to June 2016). The decline continued and reached less than 1,000 reported deaths per month in 2017.[15] A national telephone survey was conducted in April 2017 to investigate the motivations of those who continued to report deaths to the 1-1-7 system after Ebola outbreak ended.[15] Results from the survey showed that people who reported deaths were more motivated to do so when Ebola-like symptoms were present in the deceased. However, since the survey only targeted individuals who had reported a death, barriers related to reporting among those who failed to report to the 1-1-7 system were not understood. In this paper we examine death reporting barriers and explore opportunities for improving routine mortality surveillance in the aftermath of the Ebola outbreak in Sierra Leone.

Methods

In-depth qualitative interviews were conducted with 32 purposefully selected individuals who experienced a death in their household in April-August 2017 but did not report the death to the 1-1-7 system as required by the MoHS. Interviews were conducted by trained native speakers in two districts, one urban: Western Area (n=16) and one rural: Kenema district (n=16). Audio-recordings of the interviews were transcribed and the textual data were analysed using qualitative content analysis.[16] COREQ guidelines are used to describe the methods of our qualitative exploratory assessment.[17]

Setting

The initial purpose of the system when it was established in 2013 was to get feedback from communities on the government's Free Health Care Initiative that provides essential primary health care services at no-cost to children under five years old, pregnant women, and lactating mothers.[18] In August 2014, as Ebola cases began to surge, the Government repurposed the 1-1-7 line for communities to report suspected Ebola cases with a mandate that all deaths, even if not suspected of Ebola, must be reported for safe burial.[13] A national call centre was set up for triaging the call alerts to district personnel who dispatched ambulance and burial teams.[19] Alpren et al. have comprehensively documented the implementation of the 1-1-7 system.[13]

The normalization of death reporting in Sierra Leone during the Ebola outbreak presented a unique opportunity for the country to leverage the 1-1-7 system as a foundation for strengthening civil registration of vital statistics (CRVS) in the post-outbreak context. CRVS is part of global efforts to register all births and deaths occurring in all countries.[20] The 2015-2024 global strategic plan for CRVS aims to have functional country systems to record all deaths so that the WHO International Classification of Disease and Injuries

For peer review only - http://bmjopen.bmj.com/site/about/guidelines.xhtml

can be used to as "the global standard for classifying causes of death in a comparable manner over time and between populations."[20] Prior to the Ebola outbreak in Sierra Leone, deaths were supposed to be reported to the Office of Births and Deaths through its local district offices as per legal mandate established in the 1983 Births and Deaths Registration Act.[21] The pre-Ebola-outbreak death registration level remains unknown. The paper based CRVS death registration systems were never integrated with the digital platforms used in the 1-1-7 system during and after the outbreak.

The MoHS instituted enhanced surveillance mechanisms after the outbreak was declared over by WHO because it anticipated possible flare-ups of new Ebola cases due to sexual transmission of the Ebola virus by male Ebola survivors, which had been reported in Liberia.[22 23] Also, the outbreak in Guinea had not been declared over by WHO, which meant that possible importation of cases needed to be monitored, especially along border regions.[24] Consequently, the MoHS mandated that all deaths must continue to be reported to the 1-1-7 system to help detect possible flare-up of new Ebola cases. Death investigations during the enhanced surveillance period included buccal swabbing of corpses to test for Ebola and determine the need for safe burial.[13] On June 30th 2016, the MoHS announced the end of enhanced Ebola surveillance.[13] The radio announcement stated that starting in July 2016 all deaths were still required to be reported through the 1-1-7 system, however, only deaths that were suspected of Ebola were required to be investigated by district-based surveillance teams. Buccal swabbing of corpses stopped after enhanced surveillance ended. The MoHS aimed to have the 1-1-7 transitioned into the primary mechanism for death registration and mortality surveillance in Sierra Leone including for health workers to report facility-based deaths.

Sampling

Western Area and Kenema districts were purposefully selected for inclusion in the exploratory assessment. Both districts were chosen because of their epidemiological significance during the 2014-2016 Ebola outbreak in Sierra Leone and to allow for rural-urban variations in the sample. Western Area was selected because it had the highest number of reported Ebola cases and it has a large urban population. Kenema was selected because it was an early epicentre of the outbreak and it has a large rural population. We expected that the sociodemographic characteristics and the different experiences of Ebola in these districts would facilitate in-depth understanding of the range of reporting barriers and possible facilitators of willingness to report in the future. Within each district, we conducted eight interviews in high Ebola burden areas and another eight in low burden communities. Ebola epidemiological data from the MoHS showing estimated case counts at sub-district level guided our selection of communities. For this assessment, we defined high Ebola burden as $\geq$ 50 cumulative cases and low Ebola burden as $\leq$ 10 cases per community.

For peer review only - http://bmjopen.bmj.com/site/about/guidelines.xhtml

Trained data collection teams worked with community mobilizers to help identify households that had experienced one or more deaths between April and August 2017. The mobilizers contacted community leaders including religious leaders to inquire about deaths that occurred in the respective communities during the specified period. Based on the information gathered, the mobilizers and data collection teams visited the households referred by community leaders. For a referred household to be eligible to be included in the assessment, the death must not have been reported to the 1-1-7 system. We also used snowball sampling by asking eligible households to refer data collection teams to other households that may have experienced a death during the same period. All eligible households identified during recruitment agreed to participate in the assessment.

Data collection

Data collectors then continued to follow-up with the identified households to confirm eligibility, explain the purpose of the assessment, and ask for informed consent to participate. Once the eligible household was identified by the local team, informed consent was obtained from the head of the household or next of kin of the deceased. Only one interview was conducted per eligible household, and repeat interviews were not conducted. If the household head or next of kin were both unavailable, the data collection team returned to the household at least one more time before it was considered unreachable and another eligible household was approached. After obtaining informed consent from participants, the team used a structured questionnaire to gather basic demographic information about the respondent. This was followed by administering an in-depth interview using a semi-structured guide that covered two broad domains: community level practices and perceptions regarding the death; and personal experiences and perceptions regarding the death (Supplemental Material). The interview guide was pilot tested with a convenience sample of 4 respondents as part of the training of the data collection teams. Feedback from the pilot was used to improve the framing and sequencing of questions and probes.

Data collection was carried out by mixed-gender teams of interviewers and note-takers with extensive prior experience in qualitative data collection in Sierra Leone. All data collection team members were fluent in the predominant local languages of their assigned districts (Krio in Western Area, and Mende or Krio in Kenema), and had post-secondary education in social sciences or public health. All participated in a one-week training that covered informed consent, sampling procedures, best practices for conducting interviews and debriefs, and translation and transcription of audio-recordings. All interviews were audio-recorded with consent from respondents. On average the interviews lasted between 45 and 60 minutes. Interviews were conducted in a secluded area within the

For peer review only - http://bmjopen.bmj.com/site/about/guidelines.xhtml

vicinity of the home. At the end of each interview, the interviewer and note-taker conducted a short debrief that lasted approximately 30 minutes in order to capture key topics that emerged from the discussion and to document important observations that may help to contextualize the responses. Review of the data and debrief notes indicated that analytical data saturation was achieved after 16 interviews were conducted in each district (i.e. when meaningfully new information was no longer emerging from the interviews).

Data management and analysis

The respective teams of interviewers and note-takers translated and transcribed the audio-recordings. Team members conducted peer reviews of each other's transcripts to ensure consistency in translations from local language to English. A supervisor reviewed all transcripts for translation accuracy and fidelity of meaning. However, interview transcripts were not provided to the participants for their review or correction. The analysis was led by the lead author (MFJ) with support from co-author JC, both of whom are Sierra Leoneans with training and experience in qualitative data analysis. A web-based qualitative software, Dedoose®,[25] was used to support the management and analysis of the data.

An initial set of deductive codes were first generated to reflect meaning units from the questions in semi-structured interview guide. The two analysts (MFJ and JC) generated additional inductive codes from reviewing the transcripts, then proceeded to code the transcripts, independently validated each other's application of codes, and resolved any discrepancies. Textual excerpts were extracted from Dedoose® for each code. The analysts iteratively reviewed, discussed, and interpreted the coded excerpts. The final codes were organized into mutually exclusive sub-categories and categories that reflected latent grouping of concepts. An iterative, interpretative process led to the higher-level grouping of the categories into themes. Preliminary results from the qualitative analysis were presented to stakeholders in Sierra Leone including the MoHS and other surveillance partners. Feedback received from the stakeholder presentation informed our interpretation and framing of the themes.

Patient and public involvement

Patients and/or the public were not involved in the design, conduct, reporting or dissemination plans of this research.

Results

Demographic characteristics of respondents

For peer review only - http://bmjopen.bmj.com/site/about/guidelines.xhtml

Out of 32 respondents, 18 (56%) were female, 15 (47%) had no education or only primary school education, 8 (25%) were petty traders, and 22 (69%) self-identified as Muslim. Respondents' age ranged from 27 to 70 years; median age was 38 years. Respondents mostly comprised of relatives of deceased persons (29 out of 32), including their parents (n=12), spouses (n=5), children (n=4) (Table 1).

Summary of themes

Death reporting barriers were driven by the lack of awareness to report all deaths, lack of services linked to reporting (e.g. provision of ambulance services), negative experiences from the Ebola outbreak including prohibition of traditional burial rituals, perception that inevitable deaths do not need to be reported, and situations where prompt burials may be needed (Table 2). Facilitators of future willingness to report deaths were largely influenced by the perceived communicability and severity of the disease, unexplained circumstances of the death that need investigation, and the potential to leverage existing death notification practices through local leaders (Table 3). We did not observe any substantive differences in the thematic findings between the two districts and areas within districts.

Barrier 1.1. Lack of awareness to report all deaths

All respondents were unaware that they were required to report the household death after the end of the enhanced Ebola surveillance. Although some respondents knew that the 1-1-7 line was still operational, they thought that only deaths that resembled Ebola needed to be reported.

"… I do not think 1-1-7 is still existing because after Ebola we thought that was the end of 1-1-7. I'm only hearing this from you now. I always listen to the radio, but it has taken a long time I did not hear announcement that when someone dies, we are to call 1-1-7; even in the villages, that is why I did not remember to call 117." – respondent from Western Area district

Barrier 1.2 Lack of reciprocal benefit

Respondents did not see a benefit to report deaths to the 1-1-7 system in a post-Ebola-outbreak setting. The notion of simply notifying 1-1-7 without any associated follow-up action or service was not appreciated by interviewees. To report deaths to 1-1-7, respondents said they would expect some follow-up action or service to be provided. For instance, they strongly recommended for prompt ambulance services for sick people and transportation of corpses through 1-1-7. Respondents expressed that such services would help motivate them in the future to use the system.

For peer review only - http://bmjopen.bmj.com/site/about/guidelines.xhtml

"…if you take transport, like you take a taxi, to carry a [sick] person to the hospital; when you are going with him and there is traffic, they won't give you way. But let's say you call the 1-1-7 and the 1-1-7 comes with the ambulance, they will be able to give you way because they will take it as an emergency." – respondent from Western Area district

"Then I would like them to give us ambulance in the community so when someone dies, they will be able to take the person and bury him/her quickly." – respondent from Kenema district

Barrier 1.3 Perceived inevitability of certain deaths

Respondents consistently expressed that they would not consider reporting deaths that they perceive to be inevitable due to old age, God's will, and long-term illness or disability.

"The illness that affects someone for so long, for example stroke [complications], which leads to death, we will not report such death. For instance, in our community, we had a man by the name of xxx [redacted] who was affected by stroke and had suffered with it for a very long time; his family had tried all forms of medication, but he did not survive. So, with this type of death the chief themselves will just give permission to the people for burial rather than reporting to 1-1-7." - respondent from Kenema district

Barrier 1.4 Needing to bury promptly

There were two main reasons why prompt burial emerged as a barrier to death reporting. Firstly, circumstances of the death influenced perceptions of when the corpse should be buried including the death of young children, someone who has been ill for a long time (despite their age), and someone who died from an accident (e.g. drowning, fire, road accident). Secondly, Muslim respondents emphasized that they need to bury the corpse within 24 hours to honour Islamic requirements; they feared that reporting may result in burial delay based on their experiences from the Ebola outbreak. One respondent gave an example of how same-day burial was done as per Islamic tradition:

"I went straight to the Imams [at the local mosque] and notified them [of the death]. The Imam came and asked us to take the corpse to the Mosque. The corpse laid there until around twelve o'clock when they washed and wrapped it with Kasankay [white] cloth. We waited for the time … that is two o'clock, then we went to bury him. After the burial the 'Jamat' [mosque congregation] met here [at our house], ate and prayed for him before everyone went back to their homes" – respondent from Western Area district

Barrier 1.5 Negative experiences from the Ebola outbreak

For peer review only - http://bmjopen.bmj.com/site/about/guidelines.xhtml

The 1-1-7 system was intricately linked to its widespread use during the Ebola outbreak in Sierra Leone and could not be separated by respondents' negative experiences with how some deaths were handled by burial teams during the outbreak.

"The family would not get access to the corpse or even go close to it. So this knowledge had existed within people that 1-1-7 is not a call to make in order to get help in the burial of their loved ones. They will only come to do whatever they feel like, whether it is in a respectful way or not; they don't care. So, with that, people in the community do not favour the 1-1-7. Because how we expect them to bury our loved ones it's the opposite [that they will do]." – respondent from Kenema district

Most participants cited that burial methods used to bury their loved ones during the Ebola outbreak discouraged them from reporting to 1-1-7.

"This 1-1-7 line…I don't want it. I want us to be respecting the [dead] people because the 1-1-7 was not burying our people properly. So, we are burying our people. Let government leave it [burial] up to us. If a doctor check the body [that's fine], but don't let the 1-1-7 - come here until we have buried the corpse" – respondent from Kenema district

Dissatisfaction with burial methods was coupled with discontent about delays by burial teams when they were responding to death alerts during the Ebola outbreak.

"Like during the Ebola time if you call…they [burial teams] will not come [on time]. They will abandon them for some time before coming to take the person. All they care about is for the people to call them... They need to put more efforts into how they respond and treat the people with respect" – respondent from Western Area district

Fear of being quarantined was also mentioned as a barrier for reporting deaths to 1-1-7 as well as the sounds made by the ambulances and spraying of the house with chlorine, which were all associated with Ebola-related stigma. Respondents expressed that just the thought of 1-1-7 alone would usually bring back bitter memories of Ebola.

"The first time I heard about 1-1-7 was during Ebola and each time I hear about 1-1-7 I think about Ebola at once. The moment they talk about 1-1-7, it's a worry for me because during that period, when people see 1-1-7 coming, everybody would run away. When they come to a place, they will spray chlorine all over and everybody avoided body contact like nobody's business, and that worried us too much. That is why we hardly forget about 1-1-7" – respondent from Western Area district

Facilitator 1.1 Presence of Ebola-like symptoms

For peer review only - http://bmjopen.bmj.com/site/about/guidelines.xhtml

Knowledge gained during the Ebola outbreak influenced respondents' perceptions of the deaths that should be reported to the 1-1-7 system after the outbreak ended. Participants expressed that they would report a death if it resembled Ebola or Lassa fever, especially in situations where the person bled before dying.

"For any death pertaining to what government told us [we need to report]. That of a bad disease like Ebola, Lassa fever…The people around will not even dare to touch the person, because it is a transferable disease and it is more common in Eastern Province." – respondent from Kenema district

Facilitator 1.2 Sudden and unexplained deaths
Sudden and unexplained deaths wherein the person was not previously sick were perceived as needing to be reported to the authorities for further investigations.

"Like I said before, when someone dies abruptly, and nothing was wrong with him [before dying]. I will just be looking at him, I will not have the knowledge to know the cause of death, I will not have the machine to show that this is the sickness that caused the death or whether he just fell down and died or whether he just sat down and died. When you go to a medical person [through 1-1-7], he can confirm that this is the cause of the death. If the doctor has confirmed that for real he has died, what can I do?" - respondent from Western Area district

Facilitator 1.3 Existence of other local reporting mechanisms
Informing local leaders—such as religious leader, chiefs, and village headmen—about the death was a common practice that most respondents mentioned. In Kenema, some participants cited that informing local and traditional heads granted them permission for burial without needing to report to the 1-1-7 system. The reporting of these deaths to only local leaders showed that respondents were generally willing to report the deaths but only did so in localized ways outside of the 1-1-7 system.

"Well you will have to go and say to the chief or authority that someone has died amongst us. The chief will ask what happened to that person. The chief will ask you… and you will say [something like] it was a cold, or after two three days I noticed that this person was ill. God has taken his life. This is the way he died. The chief will ask you; and you should answer. You have the chance to report to the police station. You have the chance to report to the chief. And you have the chance to call the family members" – respondent from Kenema district

Discussion

For peer review only - http://bmjopen.bmj.com/site/about/guidelines.xhtml

In the aftermath of the Ebola outbreak in Sierra Leone, we identified barriers that prevented people from reporting deaths to the authorities, and we also explored facilitators that would encourage death reporting as part of routine mortality surveillance. Barriers uncovered in our assessment were linked to a lack of awareness to continue reporting all deaths after enhanced Ebola surveillance ended as well as the lack of reporting benefits. Respondents were unaware of the requirement to continue reporting all deaths after the enhanced surveillance period, and they were under the impression that only deaths resembling Ebola should be reported to the 1-1-7 system. Consequently, other deaths that were not suspected of Ebola were not reported to the 1-1-7 system. A separate assessment found that after the outbreak ended, people were more motivated to report deaths to the 1-1-7 system if Ebola-like symptoms were present in the decedent.[15] Our findings further demonstrated that although respondents did not report deaths through the 1-1-7 system as mandated by their government, they informed local councils and local leaders about the deaths. Therefore, integrating localized practices for death reporting into routine surveillance mortality systems may help optimize the number of deaths captured. Respondents complained about the lack of any reporting benefits associated with death reporting in a post-Ebola-outbreak context. They wanted ambulance services to be linked to 1-1-7 reporting as done during the outbreak. Contextually, past experiences from the Ebola outbreak served as both facilitators and barriers. Past outbreak experiences reinforced the importance of reporting when Ebola is suspected to avoid transmission risks. On the other hand, past experiences that involved dissatisfaction with how burials were handled during the outbreak may have discouraged reporting to the 1-1-7 system.

Despite efforts to promote reporting of all deaths during the outbreak, communities continued to express dissatisfaction with how their loved ones were buried and there were instances of secret burials that occurred outside of the safe burial process, which may have been due to discontent with safe burials or wanting to comply with secret society practices.[26 27] Dissatisfaction with the burial process derailed community trust to report deaths. Although dissatisfaction persisted regarding Ebola safe burial processes, communities were willing to comply with reporting because they wanted to help end Ebola transmission in the country.[28] In the waning period of the Ebola outbreak in 2015, an assessment of the community event-based surveillance showed that over 12,000 reports were submitted and investigated, out of which 287 met case-definition for suspected Ebola and 16 were confirmed positive for Ebola.[29] In that assessment, it was revealed that community event-based surveillance detected four new Ebola cases that were not epidemiologically linked and could have gone undetected.

The Ebola outbreak was tragic in many ways as demonstrated by the thousands of lives lost and the unquantifiable suffering inflicted upon the people of Sierra Leone. The pain

For peer review only - http://bmjopen.bmj.com/site/about/guidelines.xhtml

and misery they endured was evident in our assessment when they talked about their experiences during the outbreak. Even though people were sometimes dissatisfied with how the burials were handled or delayed, they recognized that it was to their benefit to have a safe burial to avoid household transmission risk. Nevertheless, normalizing death reporting during the outbreak required gaining the trust of communities by engaging them to appreciate the benefits of reporting. The halting of ambulance services after the Ebola outbreak ended prompted people to question why those services were only provided during the outbreak response.

The principle of *positive reciprocity* has been well established in social psychology,[30-32] which implies that people become motivated to comply with a request when they receive something in-return for their action. This notion is also supported in the health behaviour literature. For instance, applications of the Health Belief Model have shown that the perceived benefit associated with a behaviour is a strong predictor of engaging in the behavior.[33 34] Our findings are consistent with notions of reciprocity; respondents expressed that they will be willing to report deaths if tangible benefits are provided in return for complying with reporting.

Although it took time to establish trust between the Government and communities to achieve high level of death reporting during the Ebola outbreak,[13 35 36] communities had eventually come to expect certain services in return after reporting a death (transportation to the burial ground and laboratory testing of the corpse) and information (communication of laboratory results to the family).[13 28 35] Although similar services may not be feasible or applicable in the routine mortality surveillance environment, there is an opportunity at the community level to provide aggregated information about the deaths back to the community (e.g. through community leaders and community-based organizations) as a form of reciprocal action to foster dialogue on addressing community level health threats.[37] Another key finding from our assessment is that people want help for sick family members who are still alive. Linking the country's expanding fleet of 170 ambulances[38] with the 1-1-7 tollfree line could help promote a feeling of reciprocity in addressing other health needs in the community for people experiencing health emergencies.

Limitations

The exploratory qualitative assessment is subject to several limitations. The results are not generalizable beyond the 32 individuals interviewed. While this is an inherent limitation to most qualitative research, it is important to note that we never intended to produce generalizable results. Instead, our aim was to generate in-depth understanding of barriers and facilitators of death reporting with themes that may be transferrable to other local contexts. It is possible that some respondents may have provided socially desirable responses in terms of facilitators to report to match previously heard messages

For peer review only - http://bmjopen.bmj.com/site/about/guidelines.xhtml

during the Ebola outbreak. Because theoretical sampling was not used, as done in grounded theory approaches for example,[39] other individuals outside of family members (e.g. health workers, local city council officials) were not interviewed. Additional research with more diverse stakeholder may help shed light on additional barriers and facilitators.

Conclusion

Respondents misunderstood the *policy of reporting all deaths* after the end of enhanced Ebola surveillance in Sierra Leone, which may have been due to communication gaps in the government's official announcement of the mandate. We found that respondents perceived that only suspected Ebola deaths should be reported to the 1-1-7 system. The lack of awareness to report all deaths and the lack of perceived reporting benefits were the main reasons for failure to report the deaths. The post-outbreak death reporting policy should consider integrating community level benefits to encourage reporting. Existing practices for informal death notification through local leaders should also be leveraged to capture community-based deaths that may be missed by the formal reporting system. For example, establishing a reporting mechanism through trusted local religious and traditional leaders could help to strengthen reporting levels since these leaders are almost immediately notified of deaths that occur in their communities. Improving routine death reporting may be supported by well-planned social mobilization efforts to educate communities about the death reporting policy, promote the reporting benefits, and facilitate optimal compliance.

Acknowledgements

We thank the 32 participants for the invaluable information they provided us under difficult circumstances. This paper is dedicated to the memories of their loved ones.

Contributors

MFJ led the conceptualization and design of the assessment with primary support from JK, RK, and HN and secondary support from AJ, AEK, and REB. MFJ trained and supervised data collectors. MFJ analysed the data with support from JC and guidance from JK and HN. MFJ, JK, JC, RK, AJ, AEK, REB, and HN contributed to the interpretation of the results. MFJ led the writing of the manuscript with contributions from JK, JC, RK, AJ AEK, REB, and HN. All authors reviewed and approved the final version of the manuscript.

Funding

Data collection for this work was supported by U.S. Centers for Disease Control and Prevention through a cooperative agreement with eHealth Africa.

Competing interest

For peer review only - http://bmjopen.bmj.com/site/about/guidelines.xhtml

The authors have none to declare.

Data availability
All relevant data are presented. No additional data available.

Ethics approval
The assessment was approved by the Sierra Leone MoHS as a routine public health activity. In addition, the Center for Global Health at the U.S. Centers for Disease Control and Prevention approved the assessment as a program evaluation activity (CGH HSR Tracking # 2017-327).

Disclaimer
The findings and conclusions in this report are those of the authors and do not necessarily represent the official position of the U.S. Centers for Disease Control and Prevention, Karolinska Institutet, eHealth Africa, or Sierra Leone Ministry of Health and Sanitation.

References

1. Wauquier N, Bangura J, Moses L, et al. Understanding the emergence of ebola virus disease in sierra leone: stalking the virus in the threatening wake of emergence. *PLoS Curr* 2015;7 doi: 10.1371/currents.outbreaks.9a6530ab7bb9096b34143230ab01cdef
2. World Health Organization. Ebola situation reports, 2016. Available: http://apps.who.int/ebola/ebola-situation-reports [accessed November 27 2017].
3. Senga M, Koi A, Moses L, et al. Contact tracing performance during the Ebola virus disease outbreak in Kenema district, Sierra Leone. *Philos Trans R Soc Lond B Biol Sci* 2017;372(1721) doi: 10.1098/rstb.2016.0300 [published Online First: 2017/04/12]
4. Tiffany A, Dalziel BD, Kagume Njenge H, et al. Estimating the number of secondary Ebola cases resulting from an unsafe burial and risk factors for transmission during the West Africa Ebola epidemic. *PLoS neglected tropical diseases* 2017;11(6):e0005491. doi: 10.1371/journal.pntd.0005491 [published Online First: 2017/06/24]
5. Frieden TR, Damon IK. Ebola in West Africa--CDC's Role in Epidemic Detection, Control, and Prevention. *Emerg Infect Dis* 2015;21(11):1897-905. doi: 10.3201/eid2111.150949
6. Bedson J, Jalloh MF, Pedi D, et al. Community engagement in outbreak response: lessons from the 2014-2016 Ebola outbreak in Sierra Leone. *BMJ Glob Health* 2020
7. Pedi D, Gillespie A, Bedson J, et al. The Development of Standard Operating Procedures for Social Mobilization and Community Engagement in Sierra Leone During the West Africa Ebola Outbreak of 2014-2015. *J Health Commun* 2017;22(sup1):39-50. doi: 10.1080/10810730.2016.1212130

For peer review only - http://bmjopen.bmj.com/site/about/guidelines.xhtml

8. Setel P, AbouZahr C, Atuheire EB, et al. Mortality surveillance during the COVID-19 pandemic. *Bulletin of the World Health Organization* 2020;98(6):374. doi: 10.2471/blt.20.263194 [published Online First: 2020/06/10]
9. Michelozzi P, de'Donato F, Scortichini M, et al. Mortality impacts of the coronavirus disease (COVID-19) outbreak by sex and age: rapid mortality surveillance system, Italy, 1 February to 18 April 2020. *Euro Surveill* 2020;25(19) doi: 10.2807/1560-7917.Es.2020.25.19.2000620 [published Online First: 2020/05/21]
10. Liu S, Wu X, Lopez AD, et al. An integrated national mortality surveillance system for death registration and mortality surveillance, China. *Bulletin of the World Health Organization* 2016;94(1):46-57. doi: 10.2471/blt.15.153148 [published Online First: 2016/01/16]
11. Krishnan A, Das DK. Mortality surveillance in India: Past, present, and future. *Indian journal of public health* 2019;63(3):163-64. doi: 10.4103/ijph.IJPH_433_19 [published Online First: 2019/09/26]
12. Taylor AW, Blau DM, Bassat Q, et al. Initial findings from a novel population-based child mortality surveillance approach: a descriptive study. *Lancet Glob Health* 2020;8(7):e909-e19. doi: 10.1016/s2214-109x(20)30205-9 [published Online First: 2020/06/21]
13. Alpren C, Jalloh MF, Kaiser R, et al. The 117 call alert system in Sierra Leone: from rapid Ebola notification to routine death reporting. *BMJ Glob Health* 2017;2(3):e000392. doi: 10.1136/bmjgh-2017-000392
14. Anteneh A, Endris BS. Injury related adult deaths in Addis Ababa, Ethiopia: analysis of data from verbal autopsy. *BMC Public Health* 2020;20(1):926. doi: 10.1186/s12889-020-08944-7 [published Online First: 2020/06/17]
15. Jalloh MFK, R; Diop, M; Jambai, A; Redd, J.T.; Bunnell, R.E.; Castle, E.; Alpren, C.; Hersey, S.; Ekstrom, A.M.; Nordenstedt, H. National reporting of deaths after enhanced Ebola surveillance in Sierra Leone Freetown: Sierra Leone Ministry of Health and Sanitation, 2020.
16. Graneheim UH, Lundman B. Qualitative content analysis in nursing research: concepts, procedures and measures to achieve trustworthiness. *Nurse education today* 2004;24(2):105-12. doi: 10.1016/j.nedt.2003.10.001 [published Online First: 2004/02/11]
17. Tong A, Sainsbury P, Craig J. Consolidated criteria for reporting qualitative research (COREQ): a 32-item checklist for interviews and focus groups. *International journal for quality in health care : journal of the International Society for Quality in Health Care* 2007;19(6):349-57. doi: 10.1093/intqhc/mzm042 [published Online First: 2007/09/18]
18. Witter S, Wurie H, Bertone MP. The free health care initiative: how has it affected health workers in Sierra Leone? *Health policy and planning* 2016;31(1):1-9. doi: 10.1093/heapol/czv006 [published Online First: 2015/03/24]
19. eHealth Africa. eHealth Africa and partners boost Sierra Leone's surveillance capacity through 117 Call Center, 2019. Available: https://www.ehealthafrica.org/117-call-center [accessed February 5 2019].
20. World Bank. Global civil registration and vital statistics: Scalign up investments plan 2015-2014. Washington DC: World Bank, 2014.

For peer review only - http://bmjopen.bmj.com/site/about/guidelines.xhtml

21. United Nations Economic Commission for Africa. Births and Deaths Sierra Leone, 2012. Available: https://www.uneca.org/sites/default/files/images/birth_and_death-sierra_leone.pdf [accessed February 5 2019].
22. Deen GF, Broutet N, Xu W, et al. Ebola RNA Persistence in Semen of Ebola Virus Disease Survivors - Final Report. *N Engl J Med* 2017;377(15):1428-37. doi: 10.1056/NEJMoa1511410
23. Christie A, Davies-Wayne GJ, Cordier-Lassalle T, et al. Possible sexual transmission of Ebola virus - Liberia, 2015. *MMWR Morb Mortal Wkly Rep* 2015;64(17):479-81.
24. Diallo B, Sissoko D, Loman NJ, et al. Resurgence of Ebola Virus Disease in Guinea Linked to a Survivor With Virus Persistence in Seminal Fluid for More Than 500 Days. *Clin Infect Dis* 2016;63(10):1353-56. doi: 10.1093/cid/ciw601
25. SocioCultural Research Consultants. dedoose, 2017. Available: https://www.dedoose.com/ [accessed September 1 2017].
26. Nielsen CF, Kidd S, Sillah AR, et al. Improving burial practices and cemetery management during an Ebola virus disease epidemic - Sierra Leone, 2014. *MMWR Morb Mortal Wkly Rep* 2015;64(1):20-7.
27. Lee-Kwan SH, DeLuca N, Bunnell R, et al. Facilitators and Barriers to Community Acceptance of Safe, Dignified Medical Burials in the Context of an Ebola Epidemic, Sierra Leone, 2014. *J Health Commun* 2017;22(sup1):24-30. doi: 10.1080/10810730.2016.1209601 [published Online First: 2017/08/31]
28. Nuriddin A, Jalloh MF, Meyer E, et al. Trust, fear, stigma and disruptions: community perceptions and experiences during periods of low but ongoing transmission of Ebola virus disease in Sierra Leone, 2015. *BMJ Glob Health* 2018;3(2):e000410. doi: 10.1136/bmjgh-2017-000410 [published Online First: 2018/04/10]
29. Ratnayake R, Crowe SJ, Jasperse J, et al. Assessment of Community Event-Based Surveillance for Ebola Virus Disease, Sierra Leone, 2015. *Emerg Infect Dis* 2016;22(8):1431-7. doi: 10.3201/eid2208.160205 [published Online First: 2016/07/21]
30. Goranson REB, L. Reciprocity and responsibility reactions to prior help. *Journal of Personality and Social Psychology* 1996;3(2):227–32. doi: 10.1037/h0022895
31. Gouldner AW. The norm of reciprocity: a preliminary statement. *American Sociological Review* 1960;25(2)
32. Regan DT. Effects of a favor and liking on compliance. *Journal of Experimental Social Psychology* 1971;7(6):627-39.
33. Vermandere H, van Stam MA, Naanyu V, et al. Uptake of the human papillomavirus vaccine in Kenya: testing the health belief model through pathway modeling on cohort data. *Globalization and health* 2016;12(1):72. doi: 10.1186/s12992-016-0211-7 [published Online First: 2016/11/17]
34. Fall E, Izaute M, Chakroun-Baggioni N. How can the health belief model and self-determination theory predict both influenza vaccination and vaccination intention ? A longitudinal study among university students. *Psychology & health* 2018;33(6):746-64. doi: 10.1080/08870446.2017.1401623 [published Online First: 2017/11/15]

For peer review only - http://bmjopen.bmj.com/site/about/guidelines.xhtml

35. Li W, Jalloh MF, Bunnell R, et al. Public Confidence in the Health Care System 1 Year After the Start of the Ebola Virus Disease Outbreak - Sierra Leone, July 2015. *MMWR Morb Mortal Wkly Rep* 2016;65(21):538-42. doi: 10.15585/mmwr.mm6521a3
36. Jalloh MF, Kaiser R, Diop M, et al. National reporting of deaths after enhanced Ebola surveillance in Sierra Leone. *PLoS neglected tropical diseases* 2020;14(8):e0008624. doi: 10.1371/journal.pntd.0008624 [published Online First: 2020/08/19]
37. O'Mara Sage E, Munguambe KR, Blevins J, et al. Investigating the Feasibility of Child Mortality Surveillance With Postmortem Tissue Sampling: Generating Constructs and Variables to Strengthen Validity and Reliability in Qualitative Research. *Clin Infect Dis* 2019;69(Suppl 4):S291-s301. doi: 10.1093/cid/ciz564 [published Online First: 2019/10/11]
38. NPR. Chasing A Dream: An Ambulance Service That People Can Trust, 2018. Available: https://www.npr.org/sections/goatsandsoda/2018/08/12/637068488/chasing-a-dream-an-ambulance-service-that-people-can-trust [accessed January 28 2021].
39. Moser A, Korstjens I. Series: Practical guidance to qualitative research. Part 3: Sampling, data collection and analysis. *The European journal of general practice* 2018;24(1):9-18. doi: 10.1080/13814788.2017.1375091 [published Online First: 2017/12/05]

For peer review only

For peer review only - http://bmjopen.bmj.com/site/about/guidelines.xhtml

TABLES

Table 1. Sociodemographic characteristics of respondents, Sierra Leone, 2017

Characteristic	Number of respondents (N = 32)
**Sex**	
Female	18
Male	14
**Education**	
None or primary only	15
Secondary or higher	17
**Religion**	
Muslim	22
Christian	10
**Occupation**	
Petty trader	8
Skilled labor	7
Private business	4
Teacher	3
Student	3
Unemployed	3
Driver / bike rider	2
Farmer	1
Civil service	1
**Age (years)**	Median = 38
21-30	6
31-40	15
41-50	4
51-60	6
61-70	1
**Relationship to the deceased person**	
Parent	12
Spouse	5
Child	4
Grand parent	3
Non-relative	3
Sibling	3
Other relative	2

For peer review only - http://bmjopen.bmj.com/site/about/guidelines.xhtml

Table 2. Thematic area on barriers to reporting deaths in the aftermath of the Ebola outbreak, Sierra Leone, 2017

Code	Category	Theme
1.1.1 Reporting only required during Ebola	1.1 Lack of awareness to report all deaths	Barriers to reporting deaths in the aftermath of the Ebola outbreak
1.1.2 No more need to report to 1-1-7		
1.1.3 Only Ebola-like deaths should be reported		
1.2.1 No services for people while alive	Lack of reciprocal benefit	
1.2.2 Too much focus on dead people		
1.2.3 Nothing done for sick people		
1.2.4 Nothing happens if you report		
1.2.5 No help with burial		
1.2.6 Just a government line		
1.3.1 Old-age / God's time	1.3 Perceived inevitability of certain deaths	
1.3.2 Long-term illness or disability		
1.4.1 Islamic requirement to bury within 24 hours	1.4 Needing to bury promptly	
1.4.2 Body recovered from drowning		
1.4.3 Body recovered from fire		
1.4.4 Young child		
1.4.5 Sick for long time		
1.5.1 Wanting to forget about 117	1.5 Negative experiences from the Ebola outbreak	
1.5.2 Reporting brings sadness to family		
1.5.3 Painful memory of Ebola		
1.5.4 Burial delays during Ebola		
1.5.5 Restrictions on traditional burials		
1.5.6 Fear of quarantine		
1.5.7 Fear of ambulance		
1.5.8 Line was used during Ebola		
1.5.9 Don't like the number		

Table 3. Thematic area on facilitators of future intention to report deaths, Sierra Leone, 2017

Code	Category	Theme
1.1.1 Resembling Ebola	1.1 Presence of Ebola-like symptoms	Facilitators of willingness to report deaths in the future
1.1.2 Resembling Lassa		
1.1.3 Bleeding before dying		
1.2.1 Sudden death without illness	1.2 Sudden and unexplained death	
1.2.2 Wanting to know cause of death		
1.3.1 Informing chief	1.3 Existence of other local reporting mechanisms	
1.3.2 Informing religious leaders		
1.3.3 Informing elders		
1.3.4 Inform City Council for burial permit		
1.3.5 Informing Office of Births and Deaths for death certificate		

For peer review only - http://bmjopen.bmj.com/site/about/guidelines.xhtml

Barriers and facilitators to death reporting following Ebola surveillance in Sierra Leone: Implications for sustainable mortality surveillance

Supplemental Material: Interview Guide

Part A: Community Level Practices and Perceptions

1. **What usually happens after someone dies in this community (who does what/when/how)?**
   - Probe: What are the most important things to do immediately after someone dies?
   - Probe: How is the funeral usually handled / who participates?
   - Probe: How is the burial usually handled / who participates?
   - Probe: Who is notified of the death, and when and how are they notified?
   - Probe: To what extent does the type of death influence how the funeral/burial is handled, and who is informed about it?

2. **Who conducts/helps with the funeral/burial?**
   - Probe: Please tell us why these particular people engaged

3. **Overall, what is the current practice for reporting deaths to authorities (local, district, national 1-1-7) in this community?**
   - Probe: What types of deaths are supposed to be reported?
   - Probe: When should the death be reported, and to whom?
   - Probe: How should the death be reported?
   - Probe: What usually happens after the death is reported?

4. **What usually discourages people from reporting the death of a loved one or neighbor?**
   - Probe: is there any type of death that people are inclined not to report? What are the reasons for this?
   - Further probe for:
     - Time of death
     - Burial delay
     - Age of the deceased
     - Religious reasons
     - Other
   - Probe: What questions do you have for authorities about reporting deaths?

5. **What would encourage or motivate you to report the death of a loved one or neighbor to 117 in the future?**
   - Probe: What types of deaths are you most likely to report? What are the reasons for this?
   - Probe: What should be done differently to improve reporting?
   - Probe: What new things would you like to see happen?
   - Probe: Who can help improve death reporting in this community, if any?

Part B: Personal Experience and Perceptions

Thanks for your responses. I appreciate your openness and willingness to discuss these issues. You already told you us that you did not report the recent death of your loved one. Now, I would like to learn more about that particular situation.

For peer review only

Barriers and facilitators to death reporting following Ebola surveillance in Sierra Leone: Implications for sustainable mortality surveillance

6. Please describe for me the circumstances surrounding the death of your loved one.
- Probe: What was their health situation in the months leading up to their death?
- Probe: What signs/symptoms were reported or shown leading to the death?
- Probe: Were there suspicious aspects of the illness? If so, please describe them.
- Probe: What type of care had they received (traditional vs medical, etc)?
- Probe: Where and when did the death occur?

7. Please describe for me the steps that you or the family took after the death:
- Probe: What did you or the family first do? Why was this important?
- Probe: Who did you inform about the death? And how were they informed?
- Probe: How long did it take from time of death to the time of burial?

8. Tell us more about burial processes:
- Probe: Did you get permission for the burial? If so, how and from whom did you get approval to carry out the burial?
- Probe: Where was the body buried (public site vs private vs family land)?
- Probe: What ceremonies were conducted during the burial? (*note to interviewer: don't ask about any specific secrete ceremonies or ritual*)
- Probe: Who participated in the burial? Why were their participation important?

9. When you hear "1-1-7" what comes to mind?
- Probe: What are your opinions of 117?
- Probe: Do you think this number should be kept or change?
- Probe: Please tell us why it should be kept or changed.
- Probe: How can the 117 system be improved?

10. Please tell me the reason why you decided not to report the death to the 1-1-7 National Call Center:
- Probe: Understanding of government requirements to report all deaths to 117 (or lack thereof)?
- Probe: Concerns about funeral/burial delays?
- Probe: Nature or circumstances of the death?
  - Accident and pregnancy related
  - 'Natural causes'
  - Death that resembles Ebola or Lassa
  - Age of the deceased

11. Would you consider reporting the death of a loved one to 117 in future?
- Probe: If so please tell us what may have changed since the present case
- Probe: What types of deaths are you most likely to report? What are the reasons for this?
- Probe: What should be done differently?
- Probe: What new things would you like to see happen?
- Probe: Who can help improve death reporting in this community?

For peer review only

Barriers and facilitators to death reporting following Ebola surveillance in Sierra Leone: Implications for sustainable mortality surveillance

For peer review only

COREQ (COnsolidated criteria for REporting Qualitative research) Checklist

A checklist of items that should be included in reports of qualitative research. You must report the page number in your manuscript where you consider each of the items listed in this checklist. If you have not included this information, either revise your manuscript accordingly before submitting or note N/A.

Topic	Item No.	Guide Questions/Description	Reported on Page No.
**Domain 1: Research team and reflexivity**			
Personal characteristics			
Interviewer/facilitator	1	Which author/s conducted the interview or focus group?	
Credentials	2	What were the researcher's credentials? E.g. PhD, MD	
Occupation	3	What was their occupation at the time of the study?	
Gender	4	Was the researcher male or female?	
Experience and training	5	What experience or training did the researcher have?	
Relationship with participants			
Relationship established	6	Was a relationship established prior to study commencement?	
Participant knowledge of the interviewer	7	What did the participants know about the researcher? e.g. personal goals, reasons for doing the research	
Interviewer characteristics	8	What characteristics were reported about the inter viewer/facilitator? e.g. Bias, assumptions, reasons and interests in the research topic	
**Domain 2: Study design**			
Theoretical framework			
Methodological orientation and Theory	9	What methodological orientation was stated to underpin the study? e.g. grounded theory, discourse analysis, ethnography, phenomenology, content analysis	
Participant selection			
Sampling	10	How were participants selected? e.g. purposive, convenience, consecutive, snowball	
Method of approach	11	How were participants approached? e.g. face-to-face, telephone, mail, email	
Sample size	12	How many participants were in the study?	
Non-participation	13	How many people refused to participate or dropped out? Reasons?	
Setting			
Setting of data collection	14	Where was the data collected? e.g. home, clinic, workplace	
Presence of non-participants	15	Was anyone else present besides the participants and researchers?	
Description of sample	16	What are the important characteristics of the sample? e.g. demographic data, date	
Data collection			
Interview guide	17	Were questions, prompts, guides provided by the authors? Was it pilot tested?	
Repeat interviews	18	Were repeat inter views carried out? If yes, how many?	
Audio/visual recording	19	Did the research use audio or visual recording to collect the data?	
Field notes	20	Were field notes made during and/or after the inter view or focus group?	
Duration	21	What was the duration of the inter views or focus group?	
Data saturation	22	Was data saturation discussed?	
Transcripts returned	23	Were transcripts returned to participants for comment and/or	

For peer review only

Topic	Item No.	Guide Questions/Description	Reported on Page No.
		correction?	
**Domain 3: analysis and findings**			
Data analysis			
Number of data coders	24	How many data coders coded the data?	
Description of the coding tree	25	Did authors provide a description of the coding tree?	
Derivation of themes	26	Were themes identified in advance or derived from the data?	
Software	27	What software, if applicable, was used to manage the data?	
Participant checking	28	Did participants provide feedback on the findings?	
Reporting			
Quotations presented	29	Were participant quotations presented to illustrate the themes/findings? Was each quotation identified? e.g. participant number	
Data and findings consistent	30	Was there consistency between the data presented and the findings?	
Clarity of major themes	31	Were major themes clearly presented in the findings?	
Clarity of minor themes	32	Is there a description of diverse cases or discussion of minor themes?	

Developed from: Tong A, Sainsbury P, Craig J. Consolidated criteria for reporting qualitative research (COREQ): a 32-item checklist for interviews and focus groups. *International Journal for Quality in Health Care*. 2007. Volume 19, Number 6: pp. 349 – 357

Once you have completed this checklist, please save a copy and upload it as part of your submission. DO NOT include this checklist as part of the main manuscript document. It must be uploaded as a separate file.

For peer review only
